# Transcriptome and Metabolome Analyses in Exogenous FABP4- and FABP5-Treated Adipose-Derived Stem Cells

**DOI:** 10.1371/journal.pone.0167825

**Published:** 2016-12-09

**Authors:** Tokunori Yamamoto, Masato Furuhashi, Takeshi Sugaya, Tsuyoshi Oikawa, Megumi Matsumoto, Yasuhito Funahashi, Yoshihisa Matsukawa, Momokazu Gotoh, Tetsuji Miura

**Affiliations:** 1 Department of Urology, Nagoya University Graduate School of Medicine, Nagoya, Japan; 2 Department of Cardiovascular, Renal and Metabolic Medicine, Sapporo Medical University School of Medicine, Chuo-ku, Sapporo, Japan; 3 Department of Nephrology and Hypertension, St. Marianna University School of Medicine, Sugao, Miyamae-ku, Kawasaki, Kanagawa, Japan; 4 CIMIC Co., Ltd; Mass Building Yushima, Bunkyo-ku, Tokyo, Japan; Virgen Macarena University Hospital, School of Medicine, University of Seville, SPAIN

## Abstract

Adipose-derived stem cells (ADSC), which exist near adipocytes in adipose tissue, have been used as a potential tool of regenerative medicine. Lipid chaperones, fatty acid-binding protein 4 (FABP4) and 5 (FABP5), are abundantly expressed in adipocytes. FABP4 has recently been shown to be secreted from adipocytes during lipolysis in a non-classical pathway and may act as an adipokine. Here, we investigated the role of exogenous FABP4 and FABP5 in transcriptional and metabolic regulation in ADSC. FABP4 and FABP5 were little expressed in ADSC. However, both FABP4 and FABP5 were significantly induced after adipocyte differentiation of ADSC and were secreted from the differentiated adipocytes. Analysis of microarray data, including gene ontology enrichment analysis and cascade analysis of the protein-protein interaction network using a transcription factor binding site search, demonstrated that treatment of ADSC with FABP4 or FABP5 affected several kinds of genes related to inflammatory and metabolic responses and the process of cell differentiation. Notably, myogenic factors, including myocyte enhancer factors, myogenic differentiation 1 and myogenin, were modulated by treatment of ADSC with FABP4, indicating that exogenous FABP4 treatment is partially associated with myogenesis in ADSC. Metabolome analysis showed that treatment of ADSC with FABP4 and with FABP5 similarly, but differently in extent, promoted hydrolysis and/or uptake of lipids, consequentially together with enhancement of β oxidation, inhibition of downstream of the glycolysis pathway, accumulation of amino acids, reduction of nucleic acid components and increase in the ratio of reduced and oxidized nicotinamide adenine dinucleotide phosphates (NADPH/NADP^+^), an indicator of reducing power, and the ratio of adenosine triphosphate and adenosine monophosphate (ATP/AMP), an indicator of the energy state, in ADSC. In conclusion, secreted FABP4 and FABP5 from adipocytes as adipokines differentially affect transcriptional and metabolic regulation in ADSC near adipocytes. The adiposity condition in the host of regenerative medicine may affect characteristics of ADSC by exposure of the balance of FABP4 and FABP5.

## Introduction

Mesenchymal stem cells are multipotent somatic stem cells that can differentiate into a variety of cell types such as adipocytes, osteoblasts, chondrocytes and myocytes [1]. Mesenchymal stem cells were originally isolated from bone marrow, but they have also been isolated from other connective tissues such as adipose tissue, periosteum, synovium and deciduous teeth. A merit of regenerative medicine using autologous mesenchymal stem cells is that immunosuppression is not required. Furthermore, a large number of adipose-derived stem cells (ADSC), which exist near adipocytes in adipose tissue, can be easily obtained by liposuction, making them more clinically applicable. ADSC have therefore been used clinically as a highly potential tool of regenerative medicine.

Fatty acid-binding proteins (FABPs) are approximately 14-15-kDa predominantly cytosolic proteins that can reversibly bind saturated and unsaturated long-chain fatty acids with high affinity [2–4]. It has been proposed that intracellular FABPs facilitate the transport of lipids to specific compartments in the cell. Fatty acid-binding protein 4 (FABP4), known as adipocyte FABP (A-FABP) or aP2, and FABP5, known as epidermal FABP (E-FABP) or mal1, are expressed in both adipocytes and macrophages and play an important role in the development of insulin resistance and atherosclerosis [5–11]. We previously showed that inhibition of FABP4 in the cell would be a novel therapeutic strategy against insulin resistance, diabetes mellitus and atherosclerosis [12].

It has recently been demonstrated that FABP4 is secreted from adipocytes in association with lipolysis via a non-classical secretion pathway [13–18], though there are no typical secretory signal peptides in the sequence of FABP4 [2]. FABP4 has been demonstrated to act as an adipokine for the development of insulin resistance in the liver [14], suppression of cardiomyocyte contraction [19] and the development of atherosclerosis [20], supporting inhibition of endothelial nitric oxide synthase activity in endothelial cells [21], and proliferation and migration of vascular smooth muscle cells [22]. It has also been reported that elevated serum FABP4 concentration is associated with obesity, insulin resistance, type 2 diabetes mellitus, hypertension, cardiac dysfunction, renal dysfunction, dyslipidemia, atherosclerosis and cardiovascular events [13, 23–34]. Furthermore, a recent study demonstrated the possibility of a new strategy to treat metabolic disease by targeting serum FABP4 with a monoclonal antibody to FABP4 [35]. On the other hand, secretion of FABP5 remains to be elucidated. However, circulating FABP5, similar to FABP4, has been reported to be detected at levels of about one tenth or less of FABP4 concentrations, and FABP5 level has been shown to be associated with components of metabolic syndrome, though the correlation is not as strong as that of FABP4 [25, 36, 37].

Since FABP4 and possibly FABP5 are secreted from adipocytes near ADSC, they may play significant roles in cell survival, proliferation, migration, and transcriptional and metabolic regulation of ADSC. Depending on the condition of adiposity, such as an obese or lean condition, transcriptional and metabolic regulation and differentiation ability of ADSC would be differently modulated. Since little is known about paracrine roles of FABP4 and FABP5 derived from adipocytes in ADSC, we investigated whether exogenous FABP4 and FABP5 affect transcriptional and metabolic regulation in ADSC as well as 233A cells, another cell line of stem cells, as a control.

## Materials and Methods

### Cell culture

All biochemical reagents were purchased from Sigma-Aldrich (St. Louis, MO) unless otherwise indicated. Human ADSC were purchased from Lonza (Walkersville, MD). The 233A renal tubular stem/progenitor cell line has been established from isolated renal stem/progenitor cells isolated by primarily culturing cells exfoliated into the urine of a patient with renal disease, and the patent was issued as WO2009019758. ADSC and 233A cells were grown in MesenPRO-RS (Invitrogen, Carlsbad, CA) and Dulbecco's Modified Eagle's Medium (DMEM)/Ham’s F-12 (Invitrogen) supplemented with ITS (insulin, transferrin and selenite) mix and nicotinamide, respectively. The cells were stimulated with phosphate buffered saline as a control, 1 μM recombinant FABP4 or 1 μM recombinant FABP5 for 24 h.

### Production and purification of recombinant FABP4 and FABP5

Recombinant human FABP4 and FABP5 were produced using *Corynebacterium glutamicum*, which can extracellularly release target proteins, in a jar fermentor at 30°C for 72 h [38]. Cell-free broth was obtained by centrifugation and filtration with a 0.22-μm filter. Recombinant proteins were purified by ethanol precipitation and anion exchange chromatography. Purity of the recombinant proteins was confirmed by analyses of silver staining and Western blotting (S1 Fig).

### Western blotting and silver staining

Recombinant proteins of FABP4 (0.5 μg/lane) and FABP5 (0.3 μg/lane) and known molecular weight markers were subjected to SDS–polyacrylamide gel electrophoresis (SDS-PAGE). Proteins were electrophoretically transferred onto PVDF membranes and incubated for 1 h at room temperature with a blocking solution (3% bovine serum albumin) in Tris-buffered saline buffer containing 0.1% Tween 20 (TBST). The blocked membranes were incubated with primary antibodies for FABP4 (Abcam, Cambridge, UK) or FABP5 (Hycult, Uden, Netherlands) overnight at 4°C and washed three times with TBST. The membranes were incubated with a secondary antibody conjugated with horseradish peroxidase (Cell Signaling, Danvers, MA) for 1 h at room temperature and washed. Immunodetection analyses were performed using a BM Chemiluminescence Blotting Substrate (POD) Kit (Roche Diagnostics, Mannheim, Germany). Silver staining of gels was also performed using a SilverQuest Silver Staining kit (Thermo Fisher Scientific, Yokohama, Japan).

### Secretion of FABP4 and FABP5 from ADSC after adipocyte differentiation

Before inducing adipocyte differentiation, the conditioned medium incubated for 24 h was collected as Day 0. Adipocyte differentiation of ADSC was induced using a StemPro Adipogenesis Differentiation Kit (Invitrogen). The differentiation medium was collected at 4 days and 8 days as the conditioned media of Day 4 and Day 8, respectively. The number of samples at each time point was four. Concentrations of FABP4 and FABP5 were measured using commercially available enzyme-linked immunosorbent assay kits for FABP4 (Biovendor R&D, Modrice, Czech Republic) and FABP5 (USCN Life Science, Houston, U.S.A.). The intra- and inter-assay coefficients of variation in the kits were < 5%. According to the manufacturer’s protocol, no cross-reactivity of FABP4 or FABP5 with other FABP types was observed. Secretion of FABP4 and FABP5 into the conditioned medium was normalized to total protein concentration of the cell lysate assessed by a microplate protein assay (Bio-Rad, Hercules, CA).

### Microarray analysis of gene expression

Samples were pooled from triplicate experiments in each setup group. Total RNA from ADSC or 233A cells was extracted using an RNeasy Mini kit (Qiagen, Hilden, Germany), and RNA quality was assessed by an Agilent 2100 Bioanalyzer. Microarray analysis of gene expression was conducted using a SurePrint G3 Human GE microarray kit 8x60K (Agilent Technologies) following the Agilent 1-color microarray-based gene expression analysis protocol. Starting with 50 ng of total RNA, Cy3-labeled cRNA was produced according to the manufacturer’s protocol. For each sample, the Cy3-labeled cRNA was fragmented and hybridized at 65°C for 17 h in a rotating hybridization oven. After washing, microarrays were scanned using an Agilent microarray scanner (Agilent Technologies). Intensity values of each scanned feature were quantified using Agilent Feature Extraction Software, which performs background subtractions such as error modeling and adjusting for additive and multiplicative noise. Normalization was performed using Agilent GeneSpring GX (per chip normalization: 75 percentile shift; per gene normalization: none). The microarray data have been deposited in the NCBI Gene Expression Omnibus and are accessible through GEO series accession number GSE83587.

Gene ontology (GO) enrichment analysis was performed by Microarray Data Analysis Tool (Filgen, Nagoya, Japan). Significantly (Z-score > 0, P < 0.05) upregulated and downregulated GO terms of three GO categories, including cellular component, molecular function and biological process, were selected. A transcription factor binding site search was also carried out using the TRANSFAC database (BIOBASE, Waltham, MA) [39], and cascade analysis of the protein-protein interaction (PPI) network was performed using Genome Network Platform (http://genomenetwork.nig.ac.jp/index_e.html).

### Quantitative real-time PCR

Total RNA was isolated using Trizol Reagent (Invitrogen). One μg of total RNA was reverse-transcribed by using the high capacity cDNA archive kit (Applied Biosystems, Foster City, CA). Quantitative real-time PCR analysis was performed using SYBR Green in the real-time PCR system (Applied Biosystems, Warrington, UK). The thermal cycling program was 10 min at 95°C for enzyme activation and 40 cycles of denaturation for 15 s at 95°C, 30-s annealing at 58°C and 30-s extension at 72°C. Primers used in the present study are listed in S9 Table. To normalize expression data, 18s rRNA was used as an internal control gene. Each experiment was done in quadruplicate.

### Metabolome analysis

Comprehensive metabolite analyses in ADSC treated with phosphate buffered saline as a control, 1 μM recombinant FABP4 or 1 μM FABP5 for 24 h (n = 3, each group) were performed by capillary electrophoresis time-of-flight mass spectrometry (CE-TOFMS) and the positive and negative modes of liquid chromatography time-of-flight mass spectrometry (LC-TOFMS) as previously reported [40].

### Statistical analysis

Numeric variables are expressed as means ± SEM. Comparison between two groups was done with the Mann-Whitney U test. One-way repeated measures ANOVA was used for testing differences in time courses of parameters. One-way analysis of variance and Tukey-Kramer *post hoc* test were used for detecting significant differences in data between multiple groups. A p value of less than 0.05 was considered statistically significant. All data were analyzed by using JMP 9 for Macintosh (SAS Institute, Cary, NC).

## Results

### Effects of exogenous FABP4 on gene expression in ADSC

**Fig 1:**
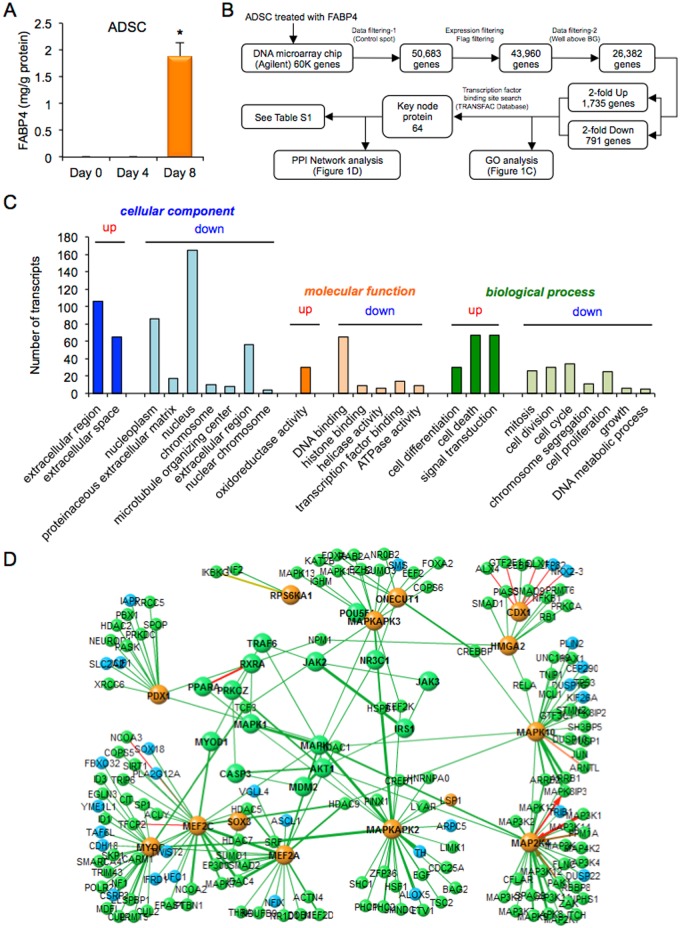
Microarray analysis in ADSC treated with FABP4. **A.** Secretion of FABP4 from adipose-derived stem cells (ADSC) after adipocyte differentiation at Day 0, Day 4 and Day 8 (n = 4 in each). Values were normalized to total protein concentration of the cell lysate. *P < 0.05 vs. Day 0. **B.** Flowchart of microarray analysis in ADSC treated with 1 μM recombinant FABP4 for 24 h. **C.** Gene ontology (GO) enrichment analysis. Significantly (Z-score > 0, P < 0.05) upregulated and downregulated GO terms of three GO categories, including cellular component, molecular function and biological process, were selected and listed by a sort of lower P-value in each category. The ordinate of the bar plot was the number of annotated genes within the GO category. **D.** Cascade of the protein-protein interaction (PPI) network using a transcription factor binding site search data.

FABP4 was not detected in the conditioned medium of ADSC at Day 0 and Day 4 after inducing adipocyte differentiation, but a high concentration of FABP4 was detected at Day 8 (Fig 1A). To determine whether secreted FABP4 has paracrine effects on gene expression in ADSC near adipocytes, DNA microarray chip analysis was performed in ADSC treated with 1 μM recombinant FABP4 for 24 h (Fig 1B–1D). A total of 26,382 genes were analyzed after quality control by removing low signal and flagged genes and control spots (Fig 1B). The numbers of genes that were at least 2-fold upregulated and at least 2-fold downregulated were 1,735 and 791, respectively.

In GO enrichment analysis, significantly upregulated and downregulated GO terms of three GO categories, including cellular component, molecular function and biological process, are shown in Fig 1C. The cellular components of upregulated genes identified by GO enrichment analysis included the extracellular region and extracellular space, while the cellular components of downregulated genes included the nucleoplasm, proteinaceous extracellular matrix, nucleus, chromosome, microtubule organizing center, extracellular region and nuclear chromosome. The molecular functions of upregulated genes included oxidoreductase activity, while the molecular functions of downregulated genes included DNA binding, histone binding, helicase activity, transcription factor binding and ATPase activity. The biological processes of upregulated genes included cell differentiation, cell death and signal transduction, while the biological processes of downregulated genes included mitosis, cell division, cell cycle, chromosome segregation, cell proliferation, growth and DNA metabolic process. These results indicated that treatment of ADSC with FABP4 affects redox, signal transduction and cell differentiation rather than cell proliferation and growth.

A transcription factor binding site search revealed that there were 64 key node proteins (Fig 1B and S1 Table). Results of cascade analysis of the PPI network are shown in Fig 1D. Key node proteins regulated by treatment of ADSC with exogenous FABP4 consisted of several kinases, including mitogen-activated protein (MAP) kinase 1 (MAPK1), also known as extracellular signal-regulated kinase 2 (ERK2), MAP kinase 10 (MAPK10), also known as c-Jun N-terminal kinase 3 (JNK3), MAP kinase-activated protein kinase 2 (MAPKAPK2), MAP kinase-activated protein kinase 3 (MAPKAPK3), dual specificity mitogen-activated protein kinase kinase 4 (MAP2K4) and ribosomal protein S6 kinase alpha 1 (RPS6KA1), and several transcription factors, including caudal type homeobox 1 (CDX1), pancreatic and duodenal homeobox 1 (PDX1), one cut homeobox 1 (ONECUT1), also known as hepatocyte nuclear factor 6 (HNF6), high mobility group AT-hook 2 (HMGA2), Pit-Oct-Unc (POU) domain class 5 homeobox 1 (POU5F1), also known as octamer-binding transcription factor 3/4 (Oct3/4), and sex-determining region Y (SRY)-box 3 (SOX3), indicating that exogenous FABP4 affects the process of cell differentiation. Nuclear receptor transcription factors, including nuclear receptor subfamily 3, group C, member 1 (NR3C1), also known as glucocorticoid receptor, peroxisome proliferator-activated receptor alpha (PPARA) and retinoid X receptor alpha (RXRA), were also involved, suggesting that treatment with FABP4 is related to metabolic response in ADSC. Furthermore, several myogenic factors, including myocyte enhancer factor 2A (MEF2A), myocyte enhancer factor 2C (MEF2C), myogenic differentiation 1 (MYOD1) and myogenin (MYOG), also known as myogenic factor 4, were modulated by treatment of ADSC with FABP4, indicating that FABP4 treatment is partially associated with myogenesis in ADSC. Gene expression of MYOD1 and MEF2A was confirmed by quantitative real-time PCR S2A and S2B Fig).

### Effects of exogenous FABP5 on gene expression in ADSC

**Fig 2:**
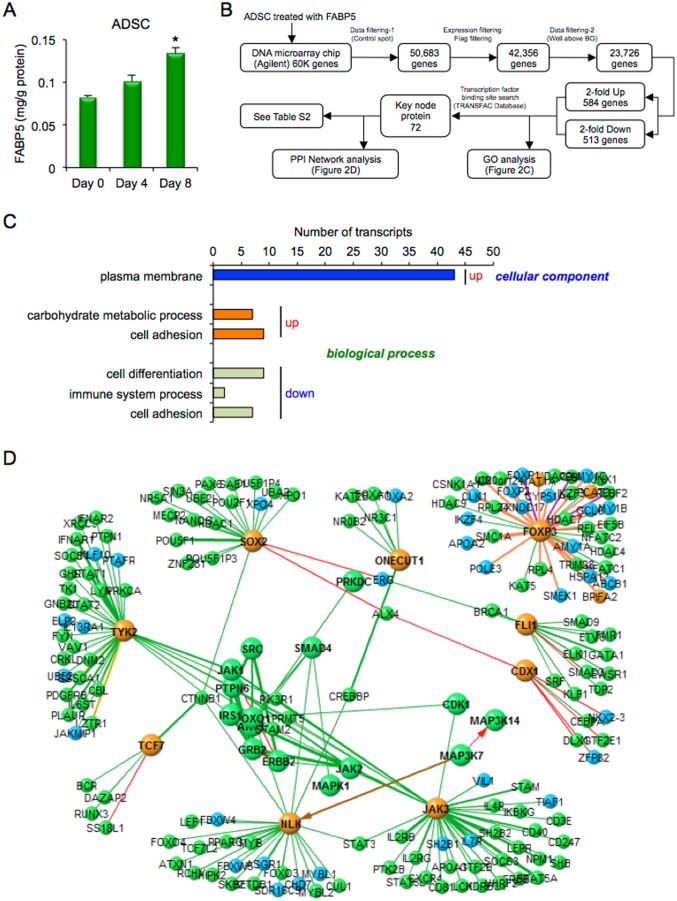
Microarray analysis in ADSC treated with FABP5. **A.** Secretion of FABP5 from adipose-derived stem cells (ADSC) after adipocyte differentiation at Day 0, Day 4 and Day 8 (n = 4 in each). Values were normalized to total protein concentration of the cell lysate. *P < 0.05 vs. Day 0. **B.** Flowchart of microarray analysis in ADSC treated with 1 μM recombinant FABP5 for 24 h. **C.** Gene ontology (GO) enrichment analysis. Significantly (Z-score > 0, P < 0.05) upregulated and downregulated GO terms of three GO categories, including cellular component, molecular function and biological process, were picked up and listed by a sort of lower P-value in each category. The abscissa of the bar plot was the number of annotated genes within the GO category. **D.** Cascade of the protein-protein interaction (PPI) network using a transcription factor binding site search data.

FABP5 was detected in the conditioned medium of ADSC at Day 0, Day 4 and Day 8 after inducing adipocyte differentiation (Fig 2A). However, the amount of FABP5 secreted at Day 8 was 14-fold smaller than that of FABP4. A total of 23,726 genes were analyzed after quality control by removing low signal and flagged genes and control spots (Fig 2B). The numbers of genes that were at least 2-fold upregulated and at least 2-fold downregulated were 584 and 513, respectively.

In GO enrichment analysis, significantly upregulated and downregulated GO terms of three GO categories, including cellular component, molecular function and biological process, are shown in Fig 2C. The cellular components of upregulated genes identified by GO enrichment analysis included the plasma membrane. The biological processes of upregulated genes included carbohydrate metabolic process and cell adhesion, while the biological processes of downregulated genes included cell differentiation, immune system process and cell adhesion. These results indicated that treatment of ADSC with FABP5 affects carbohydrate metabolism, immune response and reduced cell differentiation.

A transcription factor binding site search revealed that there were 72 key node proteins (Fig 2B and S2 Table). Results of cascade analysis of the PPI network are shown in Fig 2D. Key node proteins regulated by treatment of ADSC with exogenous FABP5 consisted of several kinases, including MAPK1, Janus kinase 3 (JAK3), tyrosine kinase 2 (TYK2) and nemo-like kinase (NLK), and several transcription factors, including CDX1, ONECUT1, SRY-box 2 (SOX2), transcription factor 7 (TCF7), friend leukemia integration 1 transcription factor (FLI1), forkhead box P3 (FOXP3), forkhead box O1 (FOXO1) and SMAD family member 4 (SMAD4), suggesting that FABP5 treatment is also associated with inflammatory response and the process of cell differentiation in ADSC. Gene expression of ONECUT1 and JAK3 was confirmed by quantitative real-time PCR (S2C and S2D Fig). The effect of treatment of ADSC with FABP4 or FABP5 was distinctly regulated in analyses of GO enrichment and the PPI network (Figs 1 and 2).

### Effects of exogenous FABP4 on gene expression in 233A cells

**Fig 3:**
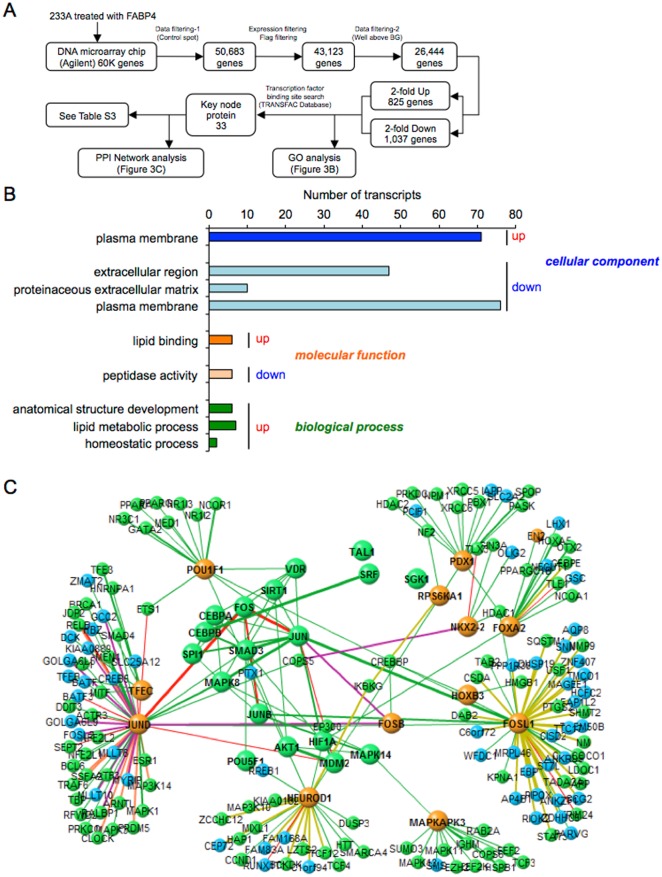
Microarray analysis in 233A cells treated with FABP4. **A.** Flowchart of microarray analysis in 233A renal tubular stem/progenitor cells treated with 1 μM recombinant FABP4 for 24 h. **B.** Gene ontology (GO) enrichment analysis. Significantly (Z-score > 0, P < 0.05) upregulated and downregulated GO terms of three GO categories, including cellular component, molecular function and biological process, were picked up and listed by a sort of lower P-value in each category. The abscissa of the bar plot was the number of annotated genes within the GO category. **C.** Cascade of the protein-protein interaction (PPI) network using a transcription factor binding site search data.

To determine paracrine effects of secreted FABP4 in another stem cell line, DNA microarray chip analysis was performed in 233A renal tubular stem/progenitor cells treated with 1 μM recombinant FABP4 for 24 h. A total of 26,444 genes were analyzed after quality control by removing low signal and flagged genes and control spots (Fig 3A). The numbers of genes that were at least 2-fold upregulated and at least 2-fold downregulated were 825 and 1,037, respectively.

In GO enrichment analysis, significantly upregulated and downregulated GO terms of three GO categories, including cellular component, molecular function and biological process, are shown in Fig 3B. The cellular components of upregulated genes identified by GO enrichment analysis included the plasma membrane, while the cellular components of downregulated genes included the extracellular region, proteinaceous extracellular matrix and plasma membrane. The molecular functions of upregulated genes included lipid binding, while the molecular functions of downregulated genes included peptidase activity. The biological processes of upregulated genes included anatomical structure development, lipid metabolic process and homeostatic process. These results indicated that treatment of 233A cells with FABP4 affects lipid binding, lipid metabolism, structure development and homeostasis.

A transcription factor binding site search revealed that there were 33 key node proteins (Fig 3A and S3 Table). Results of cascade analysis of the PPI network are shown in Fig 3C. Key node proteins regulated by treatment of 233A cells with exogenous FABP4 consisted of several kinases, including MAP kinase 8 (MAPK8), also known as c-Jun N-terminal kinase 1 (JNK1), MAPKAPK3 and RPS6KA1, and several transcription factors, including PDX1, POU1F1, NK2 homeobox 2 (NKX2-2), forkhead box F1 (FOXF1), forkhead box A2 (FOXA2), homeobox B3 (HOXB3), v-fos FBJ murine osteosarcoma viral oncogene homolog (FOS) forming the activator protein 1 (AP-1), FOS-B (FOSB), FOS-like antigen 1 (FOSL1), neurogenic differentiation 1 (NEUROD1), jun oncogene (JUN), jun B proto-oncogene (JUNB), jun D proto-oncogene (JUND), spleen focus forming virus (SFFV) proviral integration oncogene spi1 (SPI1), T-cell acute lymphocytic leukemia 1 (TAL1), SMAD family member 3 (SMAD3), transcription factor EC (TFEC), serum response factor (c-fos serum response element-binding transcription factor) (SRF), hypoxia-inducible factor 1 alpha subunit (HIF1A), CCAAT/enhancer binding protein (C/EBP) alpha (CEBPA), C/EBP beta (CEBPB), sirtuin (silent mating type information regulation 2 homolog) 1 (SIRT1) and vitamin D (1,25- dihydroxyvitamin D3) receptor (VDR). Regulated GO terms and the PPI network were greatly different for FABP4-treated ADSC and FABP4-treated 233A cells (Figs 1 and 3).

### Effects of exogenous FABP5 on gene expression in 233A cells

**Fig 4:**
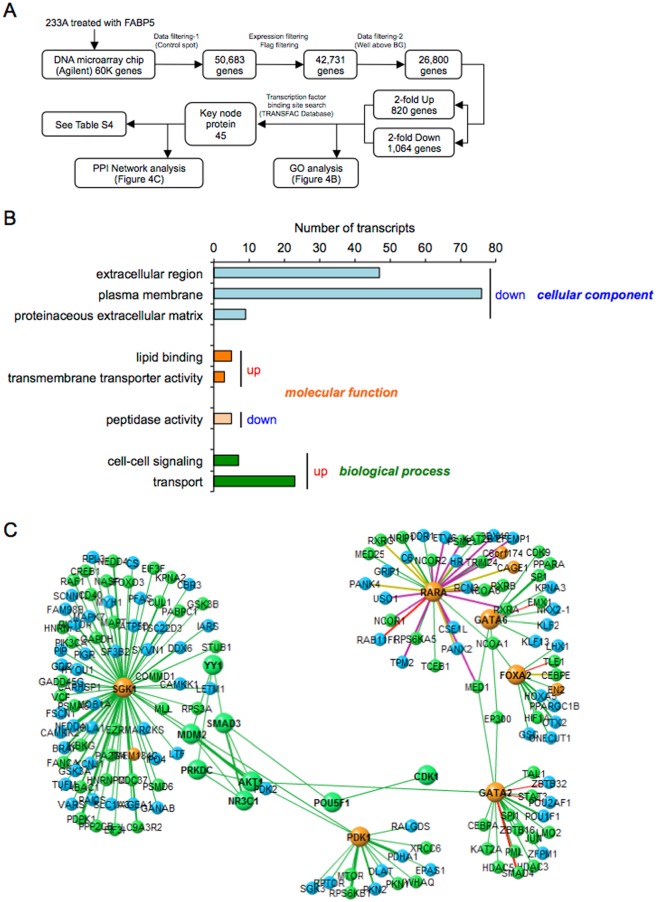
Microarray analysis in 233A cells treated with FABP5. **A.** Flowchart of microarray analysis in 233A renal tubular stem/progenitor cells treated with 1 μM recombinant FABP5 for 24 h. **B.** Gene ontology (GO) enrichment analysis. Significantly (Z-score > 0, P < 0.05) upregulated and downregulated GO terms of three GO categories, including cellular component, molecular function and biological process, were picked up and listed by a sort of lower P-value in each category. The abscissa of the bar plot was the number of annotated genes within the GO category. **C.** Cascade of the protein-protein interaction (PPI) network using a transcription factor binding site search data.

In 233A cells treated with 1 μM recombinant FABP5 for 24 h, a total of 26,800 genes were analyzed after quality control by removing low signal and flagged genes and control spots (Fig 4A). The numbers of genes that were at least 2-fold upregulated and at least 2-fold downregulated were 820 and 1,064, respectively.

In GO enrichment analysis, significantly upregulated and downregulated GO terms of three GO categories, including cellular component, molecular function and biological process, are shown in Fig 4B. The cellular components of downregulated genes included the extracellular region, plasma membrane and proteinaceous extracellular matrix. The molecular functions of upregulated genes included lipid binding and transmembrane transporter activity, while the molecular functions of downregulated genes included peptidase activity. The biological processes of upregulated genes included cell-cell signaling and transport. These results indicated that treatment of 233A cells with FABP5 affects lipid binding and transport.

A transcription factor binding site search revealed that there were 45 key node proteins (Fig 4A and S4 Table). Results of cascade analysis of the PPI network are shown in Fig 4C. Key node proteins regulated by treatment of 233A cells with exogenous FABP5 consisted of several kinases, including pyruvate dehydrogenase kinase isozyme 1 (PDK1) and serum/glucocorticoid regulated kinase 1 (SGK1), and several transcription factors, including GATA binding protein 2 (GATA2), GATA binding protein 6 (GATA6), FOXA2, retinoic acid receptor alpha (RARA), YY1 transcription factor (YY1), FOXF1, NR3C1, POU5F1 and SMAD3. Regulated GO terms and the PPI network were greatly different for FABP5-treated ADSC and FABP5-treated 233A cells (Figs 2 and 4). Furthermore, the effect of treatment of 233A cells with FABP4 or FABP5 was distinctly regulated in GO terms and the PPI network (Figs 3 and 4).

### Metabolome analysis in the treatment of ADSC with FABP4 or FABP5

**Fig 5:**
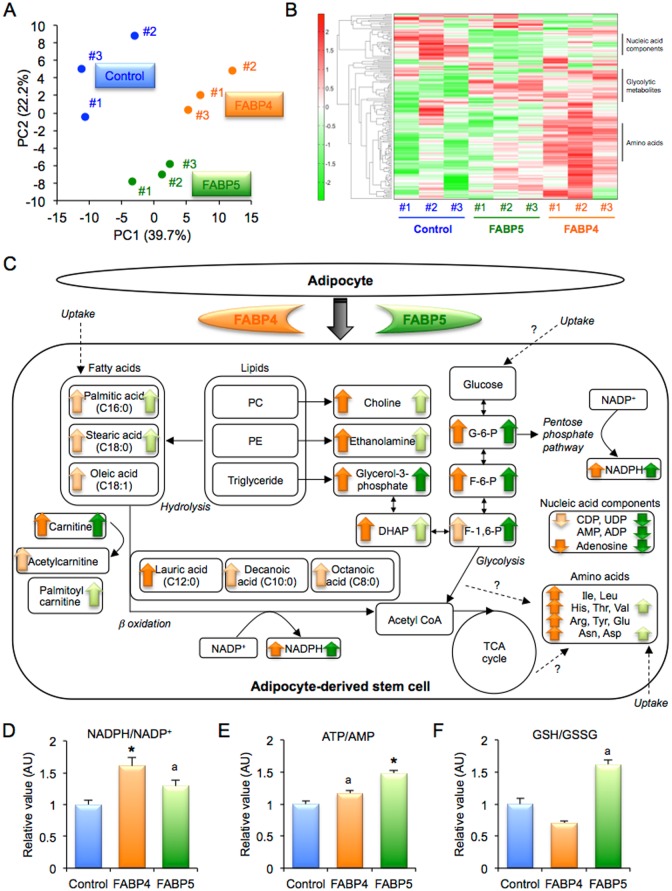
Metabolome analysis in ADSC treated with FABP4 or FABP5. **A.** Display of principal component (PC) analysis in adipose-derived stem cells (ADSC) treated with control, FABP4 and FABP5 (n = 3, each group). **B.** Heatmap display, the colors of which correspond to differences in relative abundance (low: green—high: red), demonstrates all significant alterations among treatment with control, FABP4 and FABP5. **C.** Summary scheme of regulated metabolites in ADSC treated with FABP4 or FABP5. The colors of arrows (FABP4: orange, FABP5: green) correspond to differences (dark: P < 0.05 vs. Control, light: P < 0.1). **D-F.** Relative values of metabolites including the ratio of reduced and oxidized nicotinamide adenine dinucleotide phosphates (NADPH/NADP^+^) (D), ratio of adenosine triphosphate and adenosine monophosphate (ATP/AMP) (E) and ratio of reduced and oxidized glutathione (GSH/GSSG) (F). *P < 0.05 vs. Control. ^a^P < 0.1 vs. Control. ADP, adenosine diphosphate; Arg, arginine; Asn, asparagine; Asp, aspartic acid; CDP, cytidine diphosphate; DHAP, dihydroxyacetone phosphate; F-1,6-P, fructose 1,6 diphosphate; F-6-P, fructose 6-phosphate; G-6-P, glucose 6-phosphate; Glu, glutamine; His, histidine; Ile, isoleucine; Leu, leucine; PC, phosphatidylcholine; PE, phosphatidylethanolamine; Thr, threonine; Tyr, tyrosine; UDP, uridine diphosphate; Val, valine.

In principal component (PC) analysis of metabolites, the score plot of the top two principal components, PC1 and PC2, showed that groups of ADSC treated with the control, FABP4 and FABP5 were greatly diverse (Fig 5A). A heatmap display showed that several metabolites, including nucleic acid components, glycolytic metabolites and amino acids, are distinctly regulated by treatment with FABP4 or FABP5 (Fig 5B, S5–S8 Tables). An overview map of all metabolites is shown in S3 Fig. Results for metabolites regulated by treatment with FABP4 and treatment with FABP5 are shown in S4 and S5 Figs. A summary of the effects of exogenous FABP4 and FABP5 derived from adipocytes on metabolic regulation is shown in Fig 5C.

Treatment of ADSC with FABP4 significantly increased choline, ethanolamine and glycerol 3-phosphate (S4A–S4C Fig), which are resolved from lipid components in the cell, phosphatidylcholine (PC), phosphatidylethanolamine (PE) and triglyceride, respectively. Fatty acids, palmitic acid (C16:0), stearic acid (C18:0) and oleic acid (C18:1), which are derived from hydrolysis and/or uptake of lipids, were increased by treatment with FABP4 (S4D–S4F Fig). Treatment of ADSC with FABP4 increased β oxidation-related metabolites, including carnitine, acetylcarnitine, lauric acid (C12:0), decanoic acid (C10:0) and octanoic acid (C8:0) (S4G–S4K Fig), as well as the ratio of reduced (S4L Fig) and oxidized (S4M Fig) nicotinamide adenine dinucleotide phosphates (NADPH/NADP^+^), an indicator of reducing power (Fig 5D). Intermediate products of the glycolysis pathway, including glucose 6-phosphate (G-6-P), fructose 6-phosphate (F-6-P), fructose 1,6 diphosphate (F-1,6-P) and dihydroxyacetone phosphate (DHAP), were significantly increased by treatment with FABP4 (S4N–S4Q Fig), indicating that these metabolites are induced by hydrolysis of lipids and inhibition of downstream of the glycolysis pathway rather than by uptake of glucose. Supportably, NADPH/NADP^+^, which is increased during the pentose phosphate pathway, was activated by FABP4 treatment (Fig 5D).

Treatment of ADSC with FABP4 increased several essential amino acids, including isoleucine (Ile), leucine (Leu), histidine (His), threonine (Thr) and valine (Val) (S5A–S5E Fig), suggesting an increase in uptake of these amino acids. Conditionally essential amino acids, including arginine (Arg), tyrosine (Tyr) and glutamine (Glu), and non-essential fatty acids, including asparagine (Asn) and aspartic acid (Asp), were also increased by FABP4 treatment (S5F–S5J Fig). On the other hand, nucleic acid components, including cytidine diphosphate (CDP), uridine diphosphate (UDP) and adenosine, were decreased by treatment with FABP4 (S5K–S5M Fig).

Similar results were obtained for ADSC treated with FABP5, though the extent of regulation was different (Fig 5, S4 and S5 Figs). The ratio of adenosine triphosphate and adenosine monophosphate (ATP/AMP), an indicator of the energy state, was increased in ADSC treated with FABP4 and FABP5 (Fig 5E). The ratio of reduced and oxidized glutathione (GSH/GSSG), an antioxidant indicator, was increased by treatment of ADSC with FABP5 but not with FABP4 (Fig 5F).

## Discussion

The present study demonstrated for the first time that FABP4 and FABP5 secreted from differentiated adipocytes had distinct actions of transcriptional and metabolic regulation in ADSC. Microarray analysis showed that treatment of ADSC with FABP4 or FABP5 affected several kinds of genes in relation to inflammatory and metabolic responses and the process of cell differentiation. Notably, treatment of ADSC with exogenous FABP4 was partially associated with myogenesis, suggesting that FABP4 treatment potentially has an enhancing effect on myogenesis in regenerative medicine. Furthermore, metabolome analysis showed that FABP4 and FABP5 similarly, but differently in extent, promoted hydrolysis and/or uptake of lipids, consequentially together with enhancement of β oxidation, inhibition of downstream of the glycolysis pathway, accumulation of amino acids, reduction of nucleic acid components and increase in reducing power shown by NADPH/NADP^+^ and energy state shown by ATP/AMP in ADSC.

It has been shown that obesity activates the sympathetic nerve system and induces inflammatory cytokines in adipose tissue [41]. FABP4 has been reported to be secreted from adipocytes under the condition of regulation by the lipolytic signal pathway, though FABP4 lacks an N-terminal secretory signal sequence [4, 14, 17]. Since sympathetic nerve activation and several inflammatory cytokines are known to increase lipolysis in adipocytes [42], local production of FABP4 in adipose tissue can be regulated by the condition in adipose tissue of the host, such as fat and overweight. Furthermore, expression of FABP4 and FABP5 is upregulated in a condition of increased adiposity [2–4]. Therefore, an obese condition prior to *in vivo* implantation in the host of regenerative medicine may affect the characteristics of ADSC. In addition, several therapeutic drugs, including a statin [43], omega-3 fatty acid ethyl esters [44], a dipeptidyl peptidase-4 inhibitor [45], a thiazolidinedione [46], a sodium glucose cotransporter 2 inhibitor [47] and angiotensin II receptor blockers [48, 49], have been reported to modulate circulating FABP4 level. Pretreatment with such drugs in the host before regenerative medicine may influence the effects of FABP4 and FABP5 in ADSC.

The two proteins FABP4 and FABP5 have 52% amino acid similarity and bind to various long-chain fatty acids with similar selectivity and affinity [50]. However, the expression of FABP5 is only about one-hundredth of that of FABP4 in adipose tissue [51]. Furthermore, circulating FABP5 level is detected at levels of about one tenth or less of FABP4 concentrations [25, 36, 37]. Levels of FABP4 and FABP5 in the local area around ADSC should be much higher than those in circulating blood, which are about 1 nM (15 ng/ml) for FABP4 and 0.1 nM (1.5 ng/ml) for FABP5 [25], and we therefore used recombinant FABP4 and FABP5 at the dose of 1 μM in *in vitro* experiments in the present study. However, the effects of exogenous FABP4 on ADSC might be stronger than the effects of FABP5. Exposure of the balance of FABP4 and FABP5 in ADSC dependent of the adiposity condition may differentially affect characteristics of ADSC.

We have recently been investigating whether periurethral injection of ADSC improves a damaged sphincter in patients with stress urinary incontinence (SUI) as a clinical trial registered in UMIN-CTR (UMIN000017901) and ClinicalTrials.gov (NCT02529865). Preliminary results demonstrated possible improvement of urine linkage in patients with SUI [52, 53], and we also confirmed myogenic differentiation of ADSC by periuretral injection in an SUI rat model [54]. In the present study, treatment of ADSC with exogenous FABP4, but not FABP5, was partially associated with myogenesis. Exposure of the balance of FABP4 and FABP5 in ADSC prior to *in vivo* implantation may affect muscle regeneration in patients with SUI.

FABPs have been proposed to actively facilitate the transport of lipids to specific compartments in the cell, such as the mitochondrion or peroxisome for oxidation, nucleus for lipid-mediated transcriptional regulation and endoplasmic reticulum for signaling, trafficking and membrane synthesis [2]. The present study demonstrated that not only intracellular but also exogenous FABP4 and FABP5 induce metabolic and transcriptional regulation in cells. However, regulated GO terms and the PPI network for FABP4/5-treated ADSC and FABP4/5-treated 233A cells were greatly different. There would be differences between ADSC and 233A cells in conditions that induce differentiation to a specific cell type or general differentiation rates, and such differences will provide insights into functional meanings of differences in genomic or metabolomic responses to FABPs between the two types of stem cells. Furthermore, characterization of the source of mesenchymal stem cells used in regenerative medicine, such as bone marrow, adipose tissue, periosteum, synovium and deciduous teeth, is critical.

Evidence indicating that FABP4 acts as a biological molecule is accumulating [14, 19–22, 35, 55], and serum FABP4 level has been reported to predict long-term cardiovascular events [32–34]. However, the receptor for FABP4 or FABP5 remains obscure. Furthermore, it is not known whether extracellular FABP4 and FABP5 are internalized into the cell or whether they act by an intracellular signaling mechanism. A further mechanistic understanding of the actions of FABP4 and FBAP5 may enable a promising approach of regenerative medicine using ADSC for transforming undifferentiated cells into specific cells as well as the development of new therapeutic strategies for cardiovascular and metabolic diseases, such as neutralization of FABP4 and/or blockade of the FABP4 receptor, if any.

In conclusion, secreted FABP4 and FABP5 from adipocytes as adipokines differentially affect transcriptional and metabolic regulation in ADSC near adipocytes. The adiposity condition in the host of regenerative medicine may affect characteristics of ADSC by exposure of the balance of FABP4 and FABP5.

## Supporting Information

S1 Dataset Microarray data (ADSC-FABP4). (ZIP)

S2 Dataset Microarray data (ADSC-FABP5). (ZIP)

S3 Dataset Microarray data (233A-FABP4). (ZIP)

S4 Dataset Microarray data (233A-FABP5). (ZIP)

S1 Fig Purity of recombinant FABP4 and FABP5. **A, B.** Purity of recombinant FABP4 (A) and FABP5 (B) was examined by analyses of silver staining and Western blot.
(PDF)

S2 Fig Gene expression analysis by quantitative real-time PCR. **A, B.** Gene expression of myogenic differentiation 1 (MYOD1) (A) and myocyte enhancer factor 2A (MEF2A) (B) in adipose-derived stem cells (ADSC) treated with 1 μM recombinant FABP4 for 24 h. **C, D.** Gene expression of one cut homeobox 1 (ONECUT1) (C) and Janus kinase 3 (JAK3) (D) in ADSC treated with 1 μM recombinant FABP5 for 24 h. *P < 0.05 vs. Control.
(PDF)

S3 Fig Overview map of all metabolites in metabolome analysis in ADSC treated with FABP4 or FABP5. Regulated metabolites in adipose-derived stem cells (ADSC) treated with control (blue bar), FABP4 (orange bar) and FABP5 (green bar) are shown in the map.
(PDF)

S4 Fig Metabolites of metabolome analysis in ADSC treated with FABP4 or FABP5. **A-R.** Regulated metabolites, including choline (A), ethanolamine (B), glycerol 3-phosphate (C), palmitic aicd (D), stearic acid (E), oleic acid (F), carnitine (G), acetylcarnitine (H), lauric acid (I), decanoic acid (J), octanoic acid (K), reduced nicotinamide adenine dinucleotide phosphate (NADPH) (L), oxidized nicotinamide adenine dinucleotide phosphate (NADP^+^) (M), glucose 6-phosphate (N), fructose 6-phosphate (O), fructose 1,6 diphosphate (P), dihydroxyacetone phosphate (DHAP) (Q) and palmitoylcarnitine (R), in adipose-derived stem cells (ADSC) treated with control (blue bar), FABP4 (orange bar) and FABP5 (green bar) are shown. *P < 0.05 vs. Control. ^a^P < 0.1 vs. Control.
(PDF)

S5 Fig Amino acids and nucleic acid components in metabolome analysis in ADSC treated with FABP4 or FABP5. **A-R.** Regulated metabolites, including isoleucine (Ile) (A), leucine (Leu) (B), histidine (His) (C), threonine (Thr) (D), valine (Val) (E), arginine (Arg) (F), tyrosine (Tyr) (G), glutamine (Glu) (H), asparagine (Asn) (I), aspartic acid (Asp) (J), cytidine diphosphate (CDP) (K), uridine diphosphate (UDP) (L), adenosine (M), adenosine monophosphate (AMP) (N), adenosine diphosphate (ADP) (O), adenosine triphosphate (ATP) (P), reduced glutathione (GSH) (Q) and oxidized glutathione (GSSG) (R), in adipose-derived stem cells (ADSC) treated with control (blue bar), FABP4 (orange bar) and FABP5 (green bar) are shown. *P < 0.05 vs. Control. ^a^P < 0.1 vs. Control.
(PDF)

S1 Table Key node analysis (FABP4 in ADSC). (PDF)

S2 Table Key node analysis (FABP5 in ADSC). (PDF)

S3 Table Key node analysis (FABP4 in 233A). (PDF)

S4 Table Key node analysis (FABP5 in 233A). (PDF)

S5 Table Standardized relative areas of clustering analysis by HCA (CE-TOFMS). (PDF)

S6 Table Regulated metabolites by FABP4 in ADSC (CE-TOFMS). (PDF)

S7 Table Regulated metabolites by FABP5 in ADSC (CE-TOFMS). (PDF)

S8 Table Regulated metabolites by FABP4 and FABP5 (LC-TOFMS). (PDF)

S9 Table Primers for quantitative real-time PCR. (PDF)
