# Supplementary figures and images for: Transcriptome and Metabolome Analyses in Exogenous FABP4- and FABP5-Treated Adipose-Derived Stem Cells
Source: PLoS One. 2016 Dec 9;11(12):e0167825. doi: 10.1371/journal.pone.0167825 (PMC5148007; doi:10.1371/journal.pone.0167825)

Figure S1

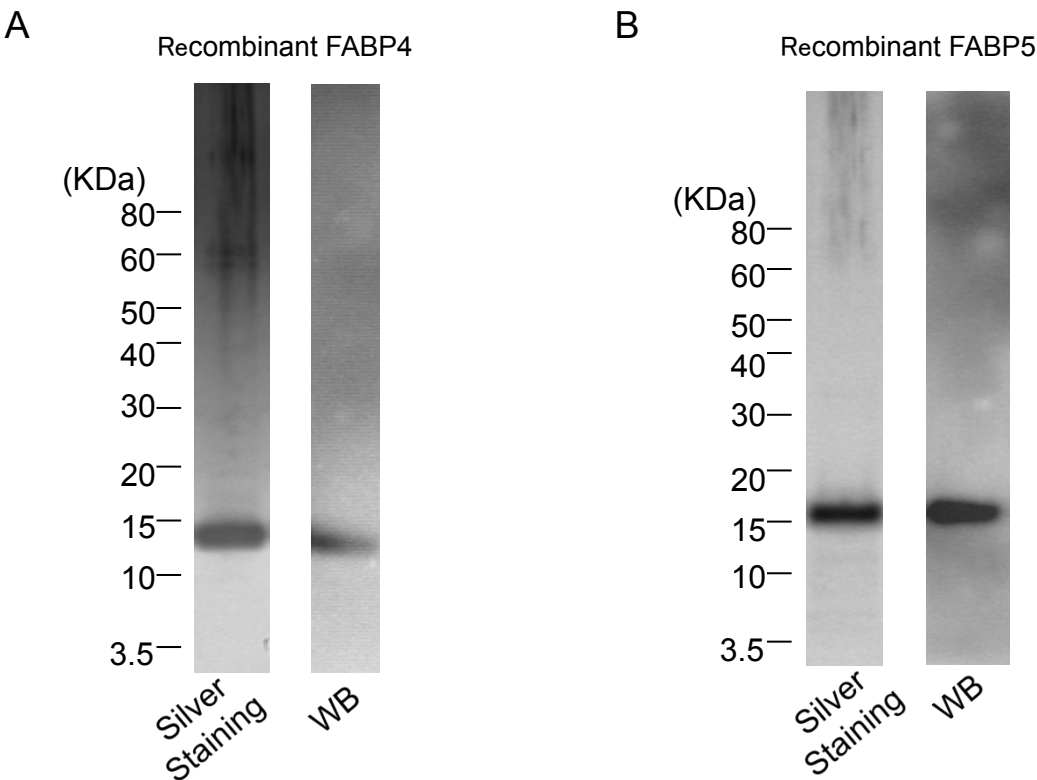

Supplement: S1 Fig — A, B. Purity of recombinant FABP4 (A) and FABP5 (B) was examined by analyses of silver staining and Western blot. (PDF) [file pone.0167825.s005.pdf]

Figure S2

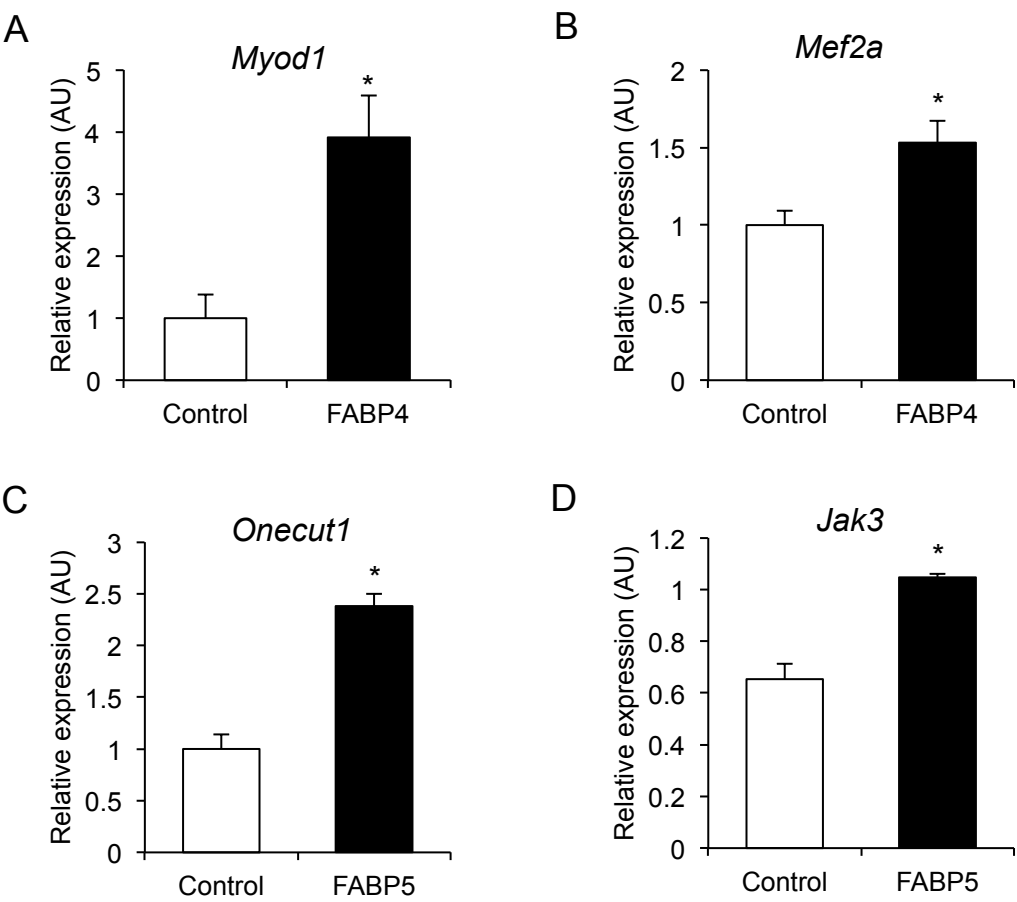

Supplement: S2 Fig — A, B. Gene expression of myogenic differentiation 1 (MYOD1) (A) and myocyte enhancer factor 2A (MEF2A) (B) in adipose-derived stem cells (ADSC) treated with 1 μM recombinant FABP4 for 24 h. C, D. Gene expression of one cut homeobox 1 (ONECUT1) (C) and Janus kinase 3 (JAK3) (D) in ADSC treated with 1 μM recombinant FABP5 for 24 h. *P < 0.05 vs. Control. (PDF) [file pone.0167825.s006.pdf]

Figure S3

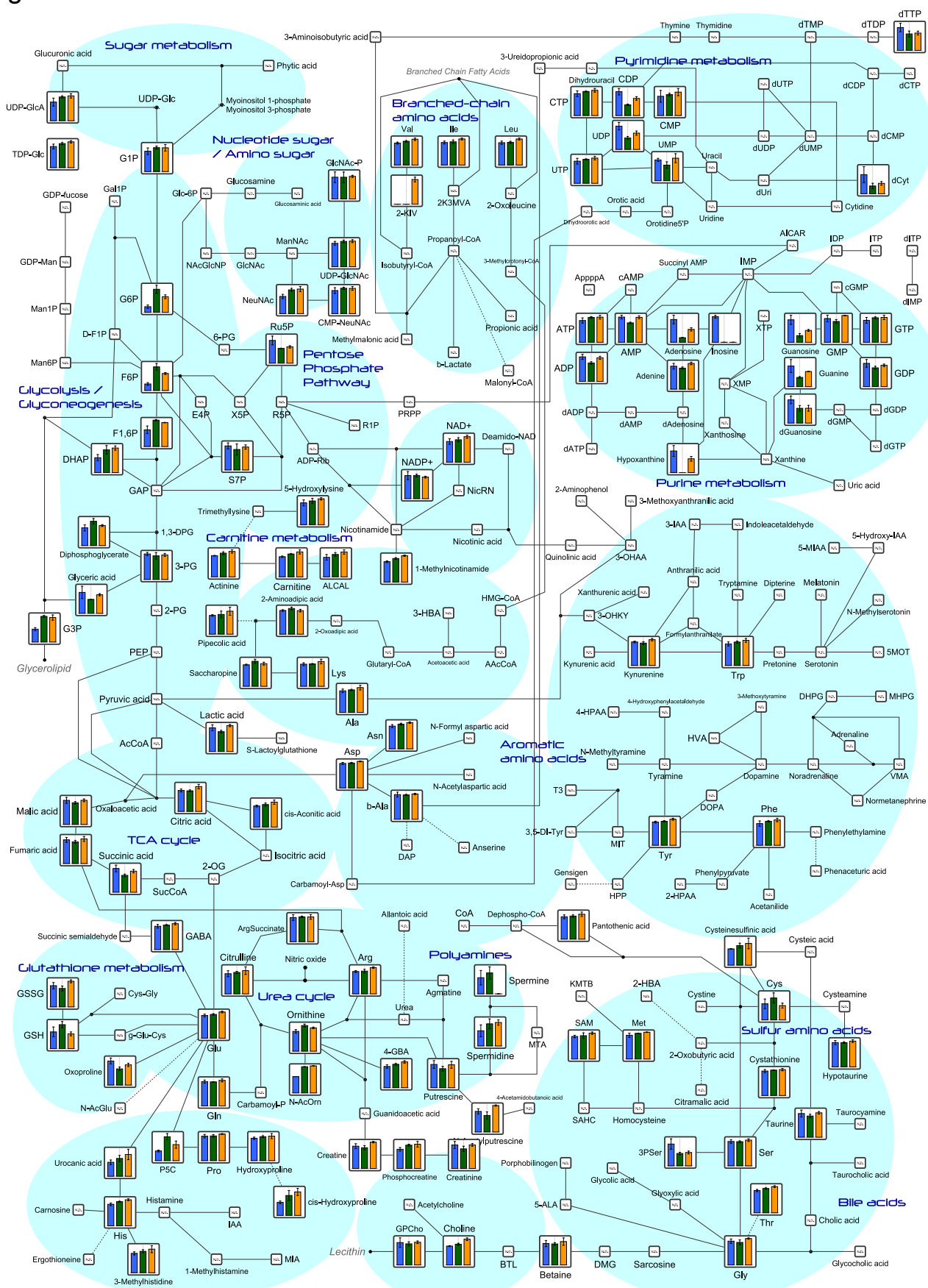

Supplement: S3 Fig — Regulated metabolites in adipose-derived stem cells (ADSC) treated with control (blue bar), FABP4 (orange bar) and FABP5 (green bar) are shown in the map. (PDF) [file pone.0167825.s007.pdf]

Figure S4

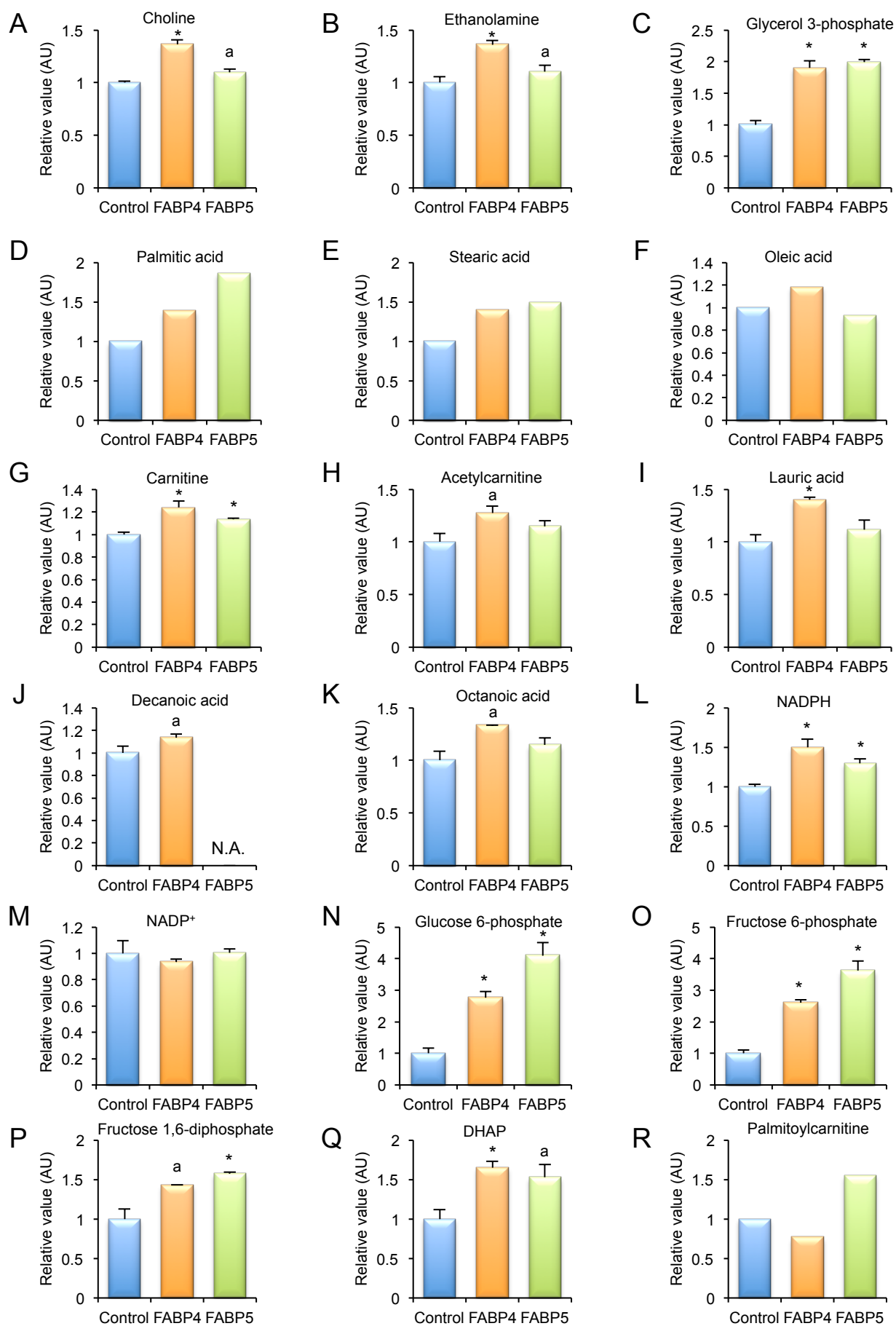

Supplement: S4 Fig — A-R. Regulated metabolites, including choline (A), ethanolamine (B), glycerol 3-phosphate (C), palmitic aicd (D), stearic acid (E), oleic acid (F), carnitine (G), acetylcarnitine (H), lauric acid (I), decanoic acid (J), octanoic acid (K), reduced nicotinamide adenine dinucleotide phosphate (NADPH) (L), oxidized nicotinamide adenine dinucleotide phosphate (NADP+) (M), glucose 6-phosphate (N), fructose 6-phosphate (O), fructose 1,6 diphosphate (P), dihydroxyacetone phosphate (DHAP) (Q) and palmitoylcarnitine (R), in adipose-derived stem cells (ADSC) treated with control (blue bar), FABP4 (orange bar) and FABP5 (green bar) are shown. *P < 0.05 vs. Control. aP < 0.1 vs. Control. (PDF) [file pone.0167825.s008.pdf]

Figure S5

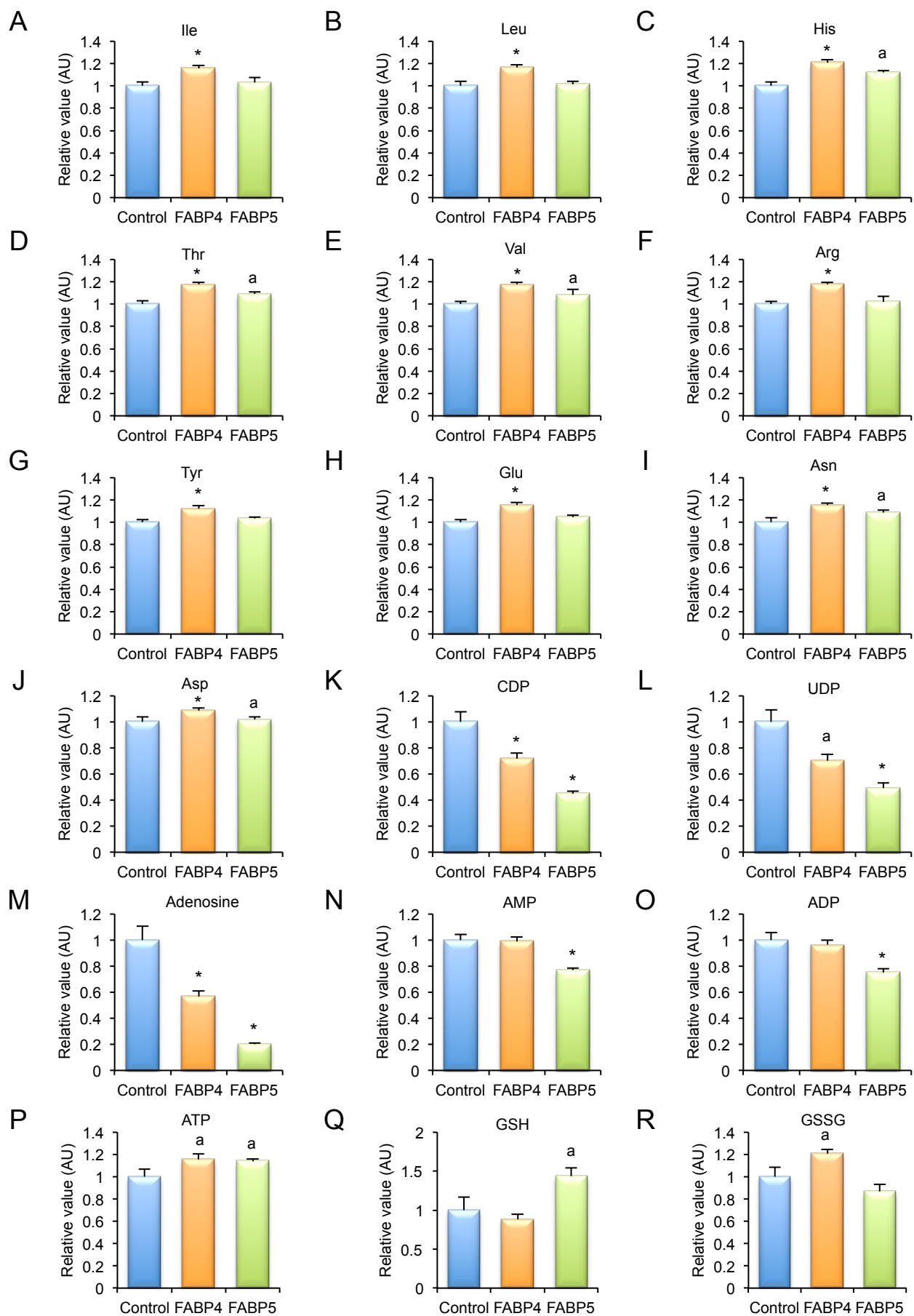

Supplement: S5 Fig — A-R. Regulated metabolites, including isoleucine (Ile) (A), leucine (Leu) (B), histidine (His) (C), threonine (Thr) (D), valine (Val) (E), arginine (Arg) (F), tyrosine (Tyr) (G), glutamine (Glu) (H), asparagine (Asn) (I), aspartic acid (Asp) (J), cytidine diphosphate (CDP) (K), uridine diphosphate (UDP) (L), adenosine (M), adenosine monophosphate (AMP) (N), adenosine diphosphate (ADP) (O), adenosine triphosphate (ATP) (P), reduced glutathione (GSH) (Q) and oxidized glutathione (GSSG) (R), in adipose-derived stem cells (ADSC) treated with control (blue bar), FABP4 (orange bar) and FABP5 (green bar) are shown. *P < 0.05 vs. Control. aP < 0.1 vs. Control. (PDF) [file pone.0167825.s009.pdf]
