# Supplementary material for: Transcriptome and Metabolome Analyses in Exogenous FABP4- and FABP5-Treated Adipose-Derived Stem Cells
Source: PLoS One. 2016 Dec 9;11(12):e0167825. doi: 10.1371/journal.pone.0167825 (PMC5148007; doi:10.1371/journal.pone.0167825)
Supplement: S1 Table — (PDF) [file pone.0167825.s010.pdf]

S1 Table

Table S1. Key node analysis (FABP4 in ADSC)

Node	Counts
IPF1	33
p38alpha	30
AKT-1	26
MAPKAPK3	25
MYOD1	25
Caspase-3	24
HMGA2	24
CKII-alpha: CKII-alpha2: (CKII-beta)2	23
JNK1alpha1	23
JNK1beta1	23
JNK2alpha-1	23
14-3-3zeta	22
ERK2	22
PKCzeta	20
MYOG	19
IRS-1	18
ERK1	17
mdm2-isoform1	17
CDX1	16
p300	16
RSK1	16
MEF2A-isoform1	15
SOX3	15
MAPKAPK2	14
CBP	13
PPARalpha	13
SHP2-isoform2	13
MEF-2C/delta8	12
MSK1	12
ONECUT1	12
JAK3	11
NR3C1	11
p/CAF	11
POU5F1	11
Src-isoform1	11
Jak2	10
JNK3alpha1	10
MKK4beta	10
p53-isoform1	10
RXR-alpha	10

Nodes (Counts <10) were omitted.
