# Supplementary material for: Transcriptome and Metabolome Analyses in Exogenous FABP4- and FABP5-Treated Adipose-Derived Stem Cells
Source: PLoS One. 2016 Dec 9;11(12):e0167825. doi: 10.1371/journal.pone.0167825 (PMC5148007; doi:10.1371/journal.pone.0167825)
Supplement: S2 Table — (PDF) [file pone.0167825.s011.pdf]

S2 Table

Table S2. Key node analysis (FABP5 in ADSC)

Node	Counts
FLI1	58
JAK2	56
p65PAK-isoform1	52
ONECUT1	51
Cdk1-isoform1	48
Grb-2-Isoform1	44
FOXO1	43
AKT-1	36
14-3-3zeta	31
FOXP3	30
IRS-1	30
SOX2	30
TCF7	30
beta-catenin	28
CDX1	28
ErbB2	24
Src-isoform1	24
DNA-PKcs-isoform1	23
IKK-beta	23
LynA	23
LynB	21
Tyk2	21
NIK	20
ERK1	19
Jak1	19
AKT1	18
SHP2-isoform2	17
ERK2	16
Smad4	16
p53beta	14
Jak3	13
SHP-1L	12
lepr-B	11
NLK	11

Nodes (Counts <10) were omitted.
