# Supplementary material for: Transcriptome and Metabolome Analyses in Exogenous FABP4- and FABP5-Treated Adipose-Derived Stem Cells
Source: PLoS One. 2016 Dec 9;11(12):e0167825. doi: 10.1371/journal.pone.0167825 (PMC5148007; doi:10.1371/journal.pone.0167825)
Supplement: S3 Table — (PDF) [file pone.0167825.s012.pdf]

# S3 Table

Table S3. Key node analysis (FABP4 in 233A)

| Node | Counts |
|---|---|
| FOXA2 | 30 |
| FOSL1 | 29 |
| CEBPB | 26 |
| JUNB | 26 |
| CEBPA | 25 |
| IPF1 | 25 |
| VDR | 22 |
| FOXF1 | 20 |
| POU5F1 | 18 |
| SPI1 | 18 |
| SRF | 18 |
| FOS | 17 |
| FOSB | 17 |
| JUND | 17 |
| mdm2-isoform1 | 17 |
| p/CAF | 17 |
| Smad3 | 17 |
| TAL1 | 16 |
| TFEC | 16 |
| MAPKAPK3 | 15 |
| POU1F1 | 15 |
| AKT-1 | 14 |
| HIF-1alpha-isoform1 | 14 |
| p53-isoform1 | 14 |
| SGK-1-isoform1 | 14 |
| 14-3-3zeta | 13 |
| JNK1alpha1 | 13 |
| RSK1 | 13 |
| SGK1 | 13 |
| HOXB3 | 12 |
| JUN | 12 |
| p300 | 12 |
| p38alpha | 12 |
| JNK1beta1 | 11 |
| NeuroD | 11 |
| NKX2-2 | 11 |

Nodes (Counts <10) were omitted.
