# Supplementary material for: Transcriptome and Metabolome Analyses in Exogenous FABP4- and FABP5-Treated Adipose-Derived Stem Cells
Source: PLoS One. 2016 Dec 9;11(12):e0167825. doi: 10.1371/journal.pone.0167825 (PMC5148007; doi:10.1371/journal.pone.0167825)
Supplement: S4 Table — (PDF) [file pone.0167825.s013.pdf]

# S4 Table

Table S4. Key node analysis (FABP5 in 233A)

| Node | Counts |
|---|---|
| FOXA2 | 36 |
| SGK-1-isoform1 | 29 |
| POU5F1 | 28 |
| p300 | 27 |
| NR3C1 | 26 |
| p/CAF | 25 |
| FOXF1 | 23 |
| AKT-1 | 22 |
| GATA2 | 20 |
| NF-IL6-1 | 20 |
| mdm2-isoform1 | 19 |
| Smad3 | 19 |
| Cdk1-isoform1 | 18 |
| SGK1 | 18 |
| YY1 | 17 |
| DNA-PKcs-isoform1 | 16 |
| p53-isoform1 | 16 |
| NR1B1-isoform1 | 14 |
| Evi-1 | 13 |
| LynA | 13 |
| CBP | 12 |
| NF-IL6-3 | 12 |
| PDK1-isoform1 | 11 |

Nodes (Counts <10) were omitted.
