# Supplementary material for: Transcriptome and Metabolome Analyses in Exogenous FABP4- and FABP5-Treated Adipose-Derived Stem Cells
Source: PLoS One. 2016 Dec 9;11(12):e0167825. doi: 10.1371/journal.pone.0167825 (PMC5148007; doi:10.1371/journal.pone.0167825)
Supplement: S5 Table — (PDF) [file pone.0167825.s014.pdf]

S5 Table

Table S5. Standardized relative areas of clustering analysis by HCA (CE-TOFMS)

	ID	Compound name	m/z	MT/RT	Control-#1	Control-#2	Control-#3	FABP5-#1	FABP5-#2	FABP5-#3	FABP4-#1	FABP4-#2	FABP4-#3
1	C_0008	Cyclohexylamine	100.11	7.64	-0.992	0.455	0.737	1.014	1.487	-0.992	-0.992	0.274	-0.992
2	C_0043	Thiaproline	134.03	13.77	-0.273	1.304	0.922	0.217	1.049	-1.270	-1.292	0.275	-0.932
3	C_0058	Penicillamine	150.06	11.74	1.018	0.694	1.278	-0.833	1.175	-0.833	-0.833	-0.833	-0.833
4	C_0029	2-Amino-2-(hydroxymethyl)-1,3-propanediol	122.08	8.18	2.213	-0.493	-0.493	-0.493	1.199	-0.493	-0.451	-0.493	-0.493
5	A_0087	NADP$^+$	742.07	9.31	2.195	-0.465	-1.114	0.703	0.408	-0.353	-0.695	-0.674	-0.004
6	C_0069	*N^5-Ethylglutamine*	*175.11*	*11.22*	0.899	1.236	-0.839	1.123	-0.839	0.939	-0.839	-0.839	-0.839
7	C_0082	β-Ala-Lys	218.15	6.78	-0.058	1.220	0.307	0.280	0.384	0.653	-1.656	-1.656	0.527
8	C_0077	Spermine	203.22	4.41	0.874	0.607	-0.406	0.298	1.431	0.696	-1.167	-1.167	-1.167
9	C_0101	Glutathione (GSH)	308.09	13.06	0.471	-0.112	-1.373	0.392	1.213	1.563	-0.744	-0.339	-1.070
10	C_0028	Cys	122.03	11.18	-0.236	0.657	-1.226	-0.218	1.146	1.735	-0.066	-0.781	-1.011
11	C_0097	Saccharopine	277.14	10.61	-0.858	-0.431	-1.001	0.763	2.081	0.084	-0.205	-0.932	0.499
12	C_0062	2-Aminoadipic acid	162.08	10.82	-0.521	0.129	-0.875	0.071	1.944	0.854	-1.488	-0.381	0.268
13	C_0033	Pipecolic acid	130.09	10.16	0.151	0.189	-0.006	0.124	-0.128	0.750	-2.465	1.087	0.299
14	A_0049	Sedoheptulose 7-phosphate	289.03	9.56	0.618	1.119	-0.448	-2.002	-0.652	1.006	-0.552	0.466	0.445
15	A_0054	UMP	323.03	9.74	0.599	0.324	-0.112	-1.458	-1.321	-0.071	-0.547	1.285	1.302
16	A_0058	AMP	346.05	9.24	1.313	0.480	0.173	-1.026	-1.467	-1.177	0.022	0.761	0.920
17	A_0025	Decanoic acid	171.14	7.88	0.658	0.635	0.307	-1.317	-1.317	-1.317	0.664	0.823	0.864
18	A_0069	ADP	426.02	10.81	0.936	1.357	-0.143	-1.238	-1.483	-0.736	0.018	0.924	0.363
19	A_0070	GDP	442.01	10.58	1.084	1.840	-0.727	-0.962	-1.254	-0.210	-0.408	0.312	0.325
20	A_0039	Ribulose 5-phosphate	229.01	10.91	1.993	1.342	0.020	-0.693	-0.793	-0.728	-0.350	-0.670	-0.121
21	A_0066	UDP	402.99	11.64	1.719	1.223	0.398	-1.213	-1.068	-0.683	-0.545	0.054	0.115
22	C_0093	Adenosine	268.10	9.78	1.657	1.142	0.617	-1.104	-1.032	-1.093	-0.257	-0.065	0.136
23	A_0065	CDP	402.01	11.42	1.171	1.600	0.549	-1.186	-1.136	-0.946	-0.260	-0.103	0.311
24	A_0014	Threonic acid	135.03	9.18	-0.139	1.481	0.835	-1.133	-0.718	-0.499	-0.572	1.477	-0.732
25	C_0055	*N*-Acetylserine	*148.06*	*22.59*	-0.223	2.145	0.634	-0.553	-0.369	0.040	-0.480	0.287	-1.480
26	A_0073	dTTP	480.98	12.10	0.683	2.137	0.130	0.210	-0.929	-1.064	-0.599	0.157	-0.725
27	C_0094	2'-Deoxyguanosine	268.10	11.40	0.363	1.115	1.876	0.012	-1.056	-0.644	-0.411	-0.071	-1.184
28	C_0095	Inosine	269.09	19.13	1.230	1.448	1.316	-0.666	-0.666	-0.666	-0.666	-0.666	-0.666
29	A_0011	5-Oxoproline	128.03	9.42	0.129	1.528	1.337	-1.530	-0.636	-0.876	-0.348	0.472	-0.075
30	C_0060	Guanine	152.06	8.20	-0.199	1.975	1.075	-1.135	-0.930	-0.862	0.001	-0.008	0.085
31	A_0010	Succinic acid	117.02	21.54	-0.106	1.739	1.027	-1.004	-1.390	-0.694	-0.384	0.476	0.336
32	C_0046	Hypoxanthine	137.04	11.01	0.375	1.418	1.172	-1.189	-1.189	-1.189	-0.034	0.199	0.438
33	C_0098	Guanosine	284.10	12.37	0.480	1.529	1.552	-0.620	-1.081	-1.120	-0.283	-0.354	-0.103
34	A_0027	*O*-Phosphoserine	*184.00*	*12.30*	0.181	2.149	1.063	-0.413	-0.867	-0.704	-0.161	-0.618	-0.629
35	C_0085	2'-Deoxycytidine	228.10	9.34	-0.282	2.054	1.226	-0.250	-1.041	-0.796	-0.087	-0.601	-0.224
36	A_0007	Glyceric acid	105.02	10.31	0.124	1.211	1.097	0.127	-1.240	-1.240	0.506	-1.240	0.655
37	A_0029	3-Phosphoglyceric acid	184.98	20.16	0.265	1.269	-0.183	0.604	-2.205	0.254	0.735	-0.066	-0.673
38	C_0003	Putrescine	89.11	4.66	-0.194	1.703	-0.192	-0.381	-1.556	-0.401	1.472	-0.103	-0.349
39	C_0056	Gln	148.08	10.62	-1.542	0.860	-0.074	-0.587	-0.298	-0.513	1.960	-0.302	0.496
40	C_0100	Glutathione (GSSG)_divalent	307.08	12.03	-1.008	-0.034	0.623	-0.149	-1.140	-1.360	1.442	0.709	0.917
41	C_0050	Urocanic acid	139.05	8.17	-1.116	-1.117	-0.274	0.670	-0.124	-0.578	2.132	0.262	0.144
42	A_0016	Octanoic acid	143.11	8.12	-1.298	0.558	0.207	0.772	0.481	-1.298	0.926	-1.298	0.951
43	C_0022	5-Aminovaleric acid	118.09	7.98	0.052	-0.849	0.000	1.409	-0.423	-0.862	0.174	-1.202	1.702
44	C_0034	Mevalolactone	131.07	21.61	-0.118	0.244	-1.697	0.753	0.724	0.571	0.658	-1.697	0.562
45	C_0099	Argininosuccinic acid	291.13	9.33	0.307	0.952	-1.919	0.309	-0.332	0.399	1.473	-0.792	-0.397
46	C_0068	*N*-Acetylornithine	*175.11*	*9.46*	0.718	-0.646	-0.646	1.541	-0.646	-0.646	1.615	-0.646	-0.646
47	A_0050	*N*-Acetylglucosamine 1-phosphate	*300.05*	*9.49*	0.585	0.883	-1.679	1.376	-0.252	-1.370	0.302	0.325	-0.169
48	A_0060	GMP	362.05	9.10	1.008	-0.006	-1.042	-0.342	-0.889	-1.534	0.860	1.017	0.929
49	C_0045	Adenine	136.06	7.56	0.765	-1.298	0.204	-0.527	-0.675	-1.328	0.549	1.489	0.821
50	A_0082	ADP-glucose<br>GDP-fucose	588.07	8.24	0.382	-0.103	-1.152	0.600	-1.777	0.388	-0.510	1.423	0.748
51	A_0053	CMP	322.04	9.52	1.219	-1.273	-1.438	0.297	-0.201	-0.302	-0.420	0.750	1.367
52	A_0046	Sorbitol 6-phosphate	261.04	9.78	1.678	-0.850	-0.912	-0.832	-0.843	0.275	-0.403	0.620	1.266
53	C_0064	5-Hydroxylysine	163.11	7.09	0.289	-0.580	-1.893	-0.756	-0.414	1.085	0.395	0.647	1.226
54	A_0041	XA0033	242.08	7.54	0.083	-0.580	-1.658	-0.075	-0.820	0.489	0.223	0.393	1.946
55	C_0073	Phosphorylcholine	184.07	20.14	0.198	-0.950	-1.736	0.114	-0.819	0.486	0.814	0.370	1.524
56	C_0096	Glu-Glu	277.10	10.74	0.449	-1.598	-1.289	1.232	-0.800	0.898	0.202	0.166	0.740
57	C_0076	11-Aminoundecanoic acid	202.18	9.60	-0.697	-0.969	-0.832	1.582	-0.307	1.466	0.118	-0.908	0.548
58	A_0047	2,3-Diphosphoglyceric acid	264.95	18.85	-0.933	0.079	-1.620	1.842	0.315	0.877	0.112	-0.322	-0.351
59	A_0042	Fructose 6-phosphate	259.02	9.89	-0.849	-1.059	-1.689	0.636	0.891	1.390	0.106	0.271	0.302
60	A_0045	Glucose 6-phosphate	259.02	9.78	-0.915	-1.203	-1.332	0.468	1.283	1.390	-0.149	0.131	0.327
61	C_0042	Ornithine	133.10	6.76	-0.417	-1.268	-1.647	0.384	1.333	1.165	0.164	0.420	-0.134
62	C_0019	1-Pyrroline 5-carboxylic acid	114.05	10.94	-0.919	-1.000	-1.185	0.604	1.539	1.195	-0.560	-0.172	0.498
63	A_0044	*myo*-Inositol 2-phosphate	*259.02*	*10.47*	-0.706	-1.202	-1.457	0.375	1.256	1.142	-0.484	0.316	0.760
64	C_0081	XC0061	218.14	9.24	-0.667	-1.176	-1.578	0.166	0.597	1.506	-0.221	0.554	0.819
65	A_0056	Fructose 1,6-diphosphate	338.99	14.69	-0.753	-0.684	-2.106	1.012	0.746	0.782	0.321	0.382	0.301
66	A_0024	Glycerol 3-phosphate	171.01	12.12	-1.261	-1.065	-1.535	0.829	0.811	0.573	0.779	0.796	0.073
67	A_0022	Dihydroxyacetone phosphate	168.99	12.70	-1.656	-0.486	-1.186	1.310	-0.012	-0.134	0.870	0.268	1.025
68	A_0051	*N*-Acetylneuraminic acid	*308.10*	*7.12*	-1.580	-0.580	-1.387	0.163	0.415	0.890	1.390	0.143	0.547
69	C_0102	XC0132	325.16	8.61	-0.797	-0.798	-1.927	-0.021	0.112	0.895	0.916	0.649	0.971
70	C_0052	γ-Butyrobetaine	146.12	8.03	-1.170	-1.111	-0.908	-0.500	0.699	0.196	1.490	0.049	1.255
71	C_0007	Glycerol	93.05	21.59	-1.873	-0.192	0.409	-1.109	1.293	0.145	-0.066	0.252	1.140
72	C_0026	Homoserine	120.07	9.98	-1.738	0.207	0.245	-1.738	0.540	0.579	0.603	0.754	0.548
73	C_0015	Diethanolamine	106.09	7.59	-1.332	0.902	-0.047	-0.698	-0.337	1.968	-0.894	0.079	0.358
74	C_0020	Creatinine	114.07	7.23	-0.853	1.442	0.664	-1.769	-0.808	-0.021	0.749	0.638	-0.041
75	C_0030	Taurine	126.02	21.55	-1.294	0.653	1.335	-1.586	-0.328	-0.363	0.143	1.112	0.328
76	C_0071	Citrulline	176.10	10.92	-1.706	-0.184	0.337	-0.217	0.381	-0.354	0.392	2.009	-0.657
77	C_0065	Methionine sulfoxide	166.05	11.65	-1.622	0.671	0.159	-0.935	0.487	-0.757	0.206	1.780	0.011
78	C_0086	Butyrylcarnitine	232.15	9.50	-2.056	1.102	-0.024	-0.778	0.321	-0.446	0.511	1.159	0.212
79	C_0014	Ser	106.05	9.89	-1.531	1.084	-0.409	-0.232	0.035	-1.029	1.197	1.272	-0.386
80	C_0091	Glycerophosphocholine	258.11	21.20	-1.051	2.092	-0.691	0.469	-0.562	-0.949	0.772	0.028	-0.108
81	A_0035	Mucic acid	209.03	14.15	-1.357	2.119	0.038	-0.203	-0.829	-0.336	0.065	0.841	-0.337
82	C_0005	β-Ala	90.06	7.27	-1.718	1.817	-0.314	-0.794	-0.064	-0.146	0.029	0.971	0.220
83	C_0016	Hypotaurine	110.03	17.79	-1.122	1.271	-1.100	-0.922	0.116	-0.235	0.247	1.713	0.033
84	A_0008	Fumaric acid	115.00	26.37	-0.612	1.371	-0.291	-1.469	-0.185	-0.513	-0.300	1.747	0.253
85	A_0013	Malic acid	133.01	21.95	-0.514	1.974	-0.313	-1.428	-0.502	-0.400	-0.137	1.107	0.214
86	A_0086	NADH	664.12	8.10	0.156	1.263	-0.956	-1.161	-0.694	-0.882	0.468	1.614	0.191
87	C_0080	Kynurenine	209.09	9.80	-0.469	0.301	-0.455	-0.664	-1.681	-0.161	0.491	1.803	0.835
88	C_0032	XC0016	129.07	8.70	-0.387	0.547	-0.354	-0.366	-2.157	0.193	0.328	1.300	0.896
89	A_0004	Lactic acid	89.02	10.71	-0.771	1.124	0.150	-1.183	-1.362	-0.578	0.513	0.903	1.205
90	C_0035	*N*-Acetylputrescine	*131.12*	*8.37*	-0.638	0.425	-0.166	-1.673	-0.994	-0.206	1.127	0.950	1.175
91	C_0002	Gly	*77.04*	8.20	-1.077	0.730	-0.216	-1.910	-0.151	-0.081	1.023	1.091	0.590
92	C_0038	Creatine	132.08	8.78	-0.512	0.624	-0.944	-1.618	-0.443	-0.320	1.139	1.260	0.813
93	C_0009	Homoserinelactone	102.06	7.05	-0.758	1.287	-1.066	-1.548	-0.551	0.379	0.843	0.908	0.505
94	C_0103	*S*-Adenosylmethionine	*399.14*	*7.04*	-0.490	-0.167	-1.214	-1.429	-0.422	1.056	1.423	0.869	0.374
95	C_0070	Arg	175.12	7.04	-0.642	-0.247	-1.121	-1.374	-0.350	0.300	1.488	1.154	0.791
96	A_0071	XA0065	445.05	6.88	-0.553	-0.359	-1.029	-0.562	-0.286	-0.800	1.327	1.889	0.375
97	C_0013	Choline	104.11	6.80	-0.824	-0.834	-1.070	-0.657	-0.041	-0.297	0.768	1.514	1.441
98	A_0009	2-Oxoisovaleric acid	115.04	10.13	-0.664	-0.664	-0.664	-0.664	-0.664	-0.664	1.103	1.434	1.444
99	A_0012	4-Methyl-2-oxovaleric acid<br>3-Methyl-2-oxovaleric acid	129.06	9.50	0.180	-0.578	-1.074	-0.763	-0.947	-0.468	0.745	1.517	1.387
100	A_0031	Citric acid	191.02	27.41	-0.891	0.206	-0.720	-0.882	-0.777	-0.202	0.099	1.898	1.269
101	C_0104	Cysteine glutathione disulfide	427.10	11.51	-1.264	-0.442	0.161	-1.134	-0.265	-0.425	0.385	1.642	1.341
102	C_0044	Asp	134.04	11.43	-1.408	-1.058	0.243	-0.959	-0.073	-0.030	1.251	1.392	0.643
103	A_0034	Lauric acid	199.17	7.57	-1.526	-0.480	-0.500	-1.107	-0.140	0.421	1.297	0.926	1.109
104	C_0074	*N*-Acetyllysine	*189.12*	*9.70*	-1.843	-1.352	-0.407	0.075	0.448	0.639	0.738	0.883	0.819
105	C_0051	4-Guanidinobutyric acid	146.09	8.18	-1.839	-0.549	-0.579	-0.132	-0.319	0.349	0.425	1.145	1.499
106	C_0010	2-Aminobutyric acid	104.07	9.55	-1.753	0.085	-1.158	0.191	-0.690	0.839	0.382	1.064	1.040
107	C_0059	Met	150.06	10.59	-1.485	0.424	-1.868	0.107	0.226	0.148	0.666	0.824	0.959
108	A_0085	NAD$^+$	662.10	6.49	-0.762	0.186	-1.738	0.403	0.098	-0.986	1.021	1.436	0.342
109	A_0079	dTDP-glucose	563.07	8.41	-1.430	0.072	-1.688	-0.039	0.641	-0.130	1.073	1.194	0.306
110	A_0020	Pelargonic acid	157.12	8.05	-1.180	-0.419	-1.460	-0.552	0.487	0.139	1.464	1.231	0.288
111	C_0061	His	156.08	7.23	-1.425	-0.441	-1.477	-0.150	0.313	0.182	1.521	0.874	0.602
112	C_0075	Gly-Asp	191.07	9.78	-1.610	-0.345	-1.446	-0.096	0.195	0.430	1.052	1.228	0.592
113	C_0041	Asn	133.06	10.36	-1.904	-0.242	-0.909	-0.417	0.530	0.151	1.042	1.319	0.430
114	C_0025	Thr	120.07	10.40	-1.570	-0.412	-1.188	-0.365	0.405	0.011	1.041	1.495	0.582
115	C_0024	Val	118.09	9.91	-1.559	-0.523	-0.924	-0.333	0.399	-0.207	1.260	1.560	0.326
116	C_0072	Tyr	182.08	11.22	-1.278	-0.139	-1.029	-0.607	0.194	-0.509	1.638	1.337	0.393
117	C_0011	GABA	104.07	7.63	-1.652	0.546	-1.222	-0.373	-0.154	0.326	1.056	1.447	0.025
118	C_0006	Ala	90.06	8.94	-1.341	0.292	-0.990	-0.453	0.086	-0.271	0.878	2.009	-0.210
119	A_0026	*cis*-Aconitic acid	*173.01*	*29.23*	-1.244	-0.406	-0.776	-0.887	0.459	-0.007	0.728	2.038	0.095
120	C_0039	Ile	132.10	10.10	-1.338	0.062	-0.781	-1.340	0.365	-0.083	1.124	1.452	0.539
121	C_0040	Leu	132.10	10.20	-1.512	-0.060	-0.362	-0.997	0.009	-0.492	1.155	1.608	0.651
122	C_0021	Pro	117.07	10.68	-1.757	0.481	-0.312	-1.253	0.418	-0.352	1.145	1.010	0.619
123	C_0057	Glu	149.06	10.79	-1.510	-0.017	-0.994	-0.750	-0.062	-0.005	0.701	1.633	1.003
124	C_0001	Ethanolamine	62.06	6.29	-0.346	-0.779	-1.453	-0.554	-0.695	0.384	1.250	1.457	0.736
125	A_0015	Ethanolamine phosphate	140.01	7.94	-0.920	-0.638	-1.595	0.061	-0.489	0.355	0.996	1.536	0.694
126	C_0047	1-Methylnicotinamide	137.07	7.29	-1.414	-0.790	-1.216	-0.251	0.023	0.377	0.951	1.378	0.942
127	C_0063	Carnitine	162.11	8.44	-0.848	-0.957	-1.379	0.154	-0.073	0.196	0.636	1.976	0.294
128	A_0061	NADPH_divalent	371.54	11.24	-0.929	-0.982	-1.370	-0.332	0.269	0.436	0.395	1.807	0.707
129	A_0038	XA0027	227.20	7.35	-0.877	-1.101	-0.713	-0.948	0.418	0.575	-0.123	1.745	1.024
130	C_0036	*cis*-4-Hydroxyproline	*132.06*	*10.83*	-1.192	-0.762	-1.169	-0.540	1.181	-0.042	0.103	1.172	1.249
131	C_0090	Isovalerylcarnitine	246.17	9.64	-1.704	-0.390	-0.485	-0.896	1.143	0.009	0.081	1.325	0.916
132	C_0078	*O*-Acetylcarnitine	*204.12*	*8.96*	-1.945	-0.503	-0.246	-0.387	0.733	-0.213	0.239	1.618	0.704
133	C_0066	Phe	166.09	10.96	-2.180	0.095	-0.541	-0.087	0.448	0.074	0.070	1.551	0.570
134	C_0079	Trp	205.10	10.88	-1.805	0.061	-0.784	-0.482	0.503	-0.001	-0.058	1.697	0.869
135	A_0080	UDP-glucose<br>UDP-galactose	565.05	8.59	-0.741	-0.353	-1.446	-0.194	0.170	0.145	-0.219	2.219	0.419
136	A_0036	Phosphocreatine	210.03	12.25	-0.913	-0.550	-1.709	0.003	0.579	0.540	0.014	1.807	0.229
137	A_0081	UDP-glucuronic acid	579.03	10.98	-0.336	-0.468	-2.259	0.368	0.653	0.060	0.478	1.318	0.186
138	A_0077	ATP	505.99	11.76	-0.257	-0.299	-2.226	0.048	0.378	0.728	-0.178	1.428	0.378
139	A_0078	GTP	521.98	11.44	0.205	-0.355	-2.197	-0.110	0.528	0.547	-0.447	1.436	0.392
140	A_0075	UTP	482.96	12.59	-0.032	0.040	-2.269	-0.086	0.007	0.334	-0.087	1.562	0.531
141	A_0074	CTP	481.98	12.35	-0.133	-0.073	-2.280	0.155	-0.033	0.352	-0.003	1.556	0.458
142	C_0083	Cystathionine	223.07	9.81	-0.420	0.453	-2.117	0.131	0.156	0.104	-0.073	1.754	0.013
143	A_0018	2-Hydroxyglutaric acid	147.03	17.28	-0.399	0.562	-1.939	-0.808	0.680	0.357	-0.215	1.561	0.202
144	A_0083	UDP-*N*-acetylglucosamine	606.07	8.41	-0.295	-0.034	-2.071	-0.525	0.491	0.989	-0.245	1.412	0.277
145	C_0053	Spermidine	146.17	4.47	-1.014	0.195	-1.902	-0.655	0.649	1.006	0.221	1.122	0.378
146	A_0084	CMP-*N*-acetylneuraminate	613.14	8.09	-0.722	0.665	-2.056	-0.194	0.634	0.737	0.358	1.164	-0.586
147	C_0092	Thiamine	265.11	6.54	-0.498	1.033	-2.023	-0.404	0.698	0.815	0.486	0.667	-0.772
148	A_0043	Glucose 1-phosphate	259.02	10.08	-1.363	0.485	-1.460	0.331	-0.039	0.945	1.098	0.887	-0.883
149	C_0037	Hydroxyproline	132.06	11.89	-1.357	-0.093	-0.703	0.709	-0.363	0.208	1.813	0.807	-1.020
150	A_0037	Pantothenic acid	218.10	7.59	-1.443	0.474	-0.736	0.201	-0.902	0.111	1.683	1.101	-0.489
151	C_0067	3-Methylhistidine	170.09	7.42	-1.389	-0.320	-0.550	-0.519	-0.152	0.629	1.826	1.112	-0.636
152	C_0054	Lys	147.11	6.82	-0.685	0.299	-1.188	-0.538	-0.459	-0.160	1.845	1.367	-0.480
153	A_0032	Gluconic acid	195.05	8.09	-0.192	0.863	-0.508	-0.909	-0.544	-0.366	1.451	1.439	-1.235
154	C_0023	Betaine	118.09	11.14	0.283	0.621	-1.469	-0.880	-0.013	-0.139	0.668	1.826	-0.898
155	A_0019	Cysteinesulfinic acid	152.00	9.54	0.208	0.214	0.146	-1.660	0.379	0.759	0.382	1.231	-1.660
