# Supplementary material for: Transcriptome and Metabolome Analyses in Exogenous FABP4- and FABP5-Treated Adipose-Derived Stem Cells
Source: PLoS One. 2016 Dec 9;11(12):e0167825. doi: 10.1371/journal.pone.0167825 (PMC5148007; doi:10.1371/journal.pone.0167825)
Supplement: S6 Table — (PDF) [file pone.0167825.s015.pdf]

S6 Table

Table S6. Regulated metabolites by FABP4 in ADSC (CE-TOFMS)

ID	Compound name	Ratio	P
C_0047	1-Methylnicotinamide	1.3	0.001
A_0045	Glucose 6-phosphate	2.8	0.003
A_0015	Ethanolamine phosphate	1.2	0.005
A_0024	Glycerol 3-phosphate	1.9	0.006
C_0070	Arg	1.2	0.006
C_0013	Choline	1.4	0.007
C_0061	His	1.2	0.009
C_0001	Ethanolamine	1.4	0.010
C_0025	Thr	1.2	0.010
A_0020	Pelargonic acid	1.4	0.014
C_0024	Val	1.2	0.014
A_0022	Dihydroxyacetone phosphate	1.6	0.015
C_0026	Homoserine	1.2	0.016
A_0042	Fructose 6-phosphate	2.6	0.018
A_0051	*N*-Acetylneuraminic acid	1.4	0.018
C_0072	Tyr	1.1	0.019
C_0075	Gly-Asp	1.1	0.020
C_0081	XC0061	1.7	0.021
C_0051	4-Guanidinobutyric acid	1.2	0.022
A_0034	Lauric acid	1.4	0.022
A_0012	4-Methyl-2-oxovaleric acid 3-Methyl-2-oxovaleric acid	1.5	0.023
C_0036	*cis*-4-Hydroxyproline	1.8	0.025
C_0103	*S*-Adenosylmethionine	1.1	0.025
C_0057	Glu	1.2	0.027
C_0102	XC0132	1.3	0.027
C_0039	Ile	1.2	0.030
A_0061	NADPH_divalent	1.5	0.031
C_0040	Leu	1.2	0.035
A_0071	XA0065	1.2	0.036
C_0041	Asn	1.2	0.036
C_0074	*N*-Acetyllysine	1.3	0.040
C_0094	2'-Deoxyguanosine	0.6	0.041
C_0052	γ-Butyrobetaine	1.2	0.043
C_0093	Adenosine	0.6	0.044
C_0104	Cysteine glutathione disulfide	1.6	0.044
C_0090	Isovalerylcarnitine	1.2	0.044
A_0065	CDP	0.7	0.046
A_0044	*myo*-Inositol 2-phosphate	1.4	0.047
C_0063	Carnitine	1.2	0.048
C_0044	Asp	1.1	0.050
