# Supplementary material for: Transcriptome and Metabolome Analyses in Exogenous FABP4- and FABP5-Treated Adipose-Derived Stem Cells
Source: PLoS One. 2016 Dec 9;11(12):e0167825. doi: 10.1371/journal.pone.0167825 (PMC5148007; doi:10.1371/journal.pone.0167825)
Supplement: S7 Table — (PDF) [file pone.0167825.s016.pdf]

S7 Table

Table S7. Regulated metabolites by FABP5 in ADSC (CE-TOFMS)

ID	Compound name	Ratio	P
A_0024	Glycerol 3-phosphate	2.0	0.001
A_0044	*myo*-Inositol 2-phosphate	1.7	0.005
A_0042	Fructose 6-phosphate	3.6	0.006
C_0026	Homoserine	1.2	0.007
C_0063	Carnitine	1.1	0.008
A_0045	Glucose 6-phosphate	4.1	0.009
C_0047	1-Methylnicotinamide	1.2	0.010
C_0019	1-Pyrroline 5-carboxylic acid	2.5	0.011
C_0042	Ornithine	1.3	0.013
A_0051	*N*-Acetylneuraminic acid	1.4	0.014
C_0098	Guanosine	0.3	0.014
A_0065	CDP	0.4	0.015
A_0061	NADPH_divalent	1.3	0.017
C_0093	Adenosine	0.2	0.017
A_0066	UDP	0.5	0.020
A_0058	AMP	0.8	0.021
C_0081	XC0061	1.8	0.021
A_0011	5-Oxoproline	0.7	0.025
A_0036	Phosphocreatine	1.2	0.033
A_0069	ADP	0.8	0.035
C_0102	XC0132	1.2	0.037
C_0094	2'-Deoxyguanosine	0.6	0.040
A_0056	Fructose 1,6-diphosphate	1.6	0.044
C_0074	*N*-Acetyllysine	1.3	0.049
