# Supplementary material for: Transcriptome and Metabolome Analyses in Exogenous FABP4- and FABP5-Treated Adipose-Derived Stem Cells
Source: PLoS One. 2016 Dec 9;11(12):e0167825. doi: 10.1371/journal.pone.0167825 (PMC5148007; doi:10.1371/journal.pone.0167825)
Supplement: S8 Table — (PDF) [file pone.0167825.s017.pdf]

S8 Table

Table S8. Regulated metabolites by FABP4 and FABP5 (LC-TOFMS)

ID	Compound name	Ratio	
		FABP4	FABP5
P_0005	Oleoyl ethanolamide	1.3	1.1
P_0017	Trilaurin	0.9	1.0
P_0006	Stearoyl ethanolamide	0.9	1.0
N_0008	Stearic acid	1.4	1.5
N_0004	Palmitic acid	1.4	1.9
P_0003	Palmitoylethanolamide	0.9	1.0
N_0024	1-Palmitoyl-glycero-3-phosphoethanolamine	1.2	1.1
P_0014	1-Oleoyl-glycero-3-phosphocholine	1.3	0.8
P_0021	1,2-Distearoyl-glycero-3-phosphocholine	0.7	1.0
P_0010	Palmitoylcarnitine	0.8	1.6
P_0002	Sphingosine	0.9	0.8
P_0015	1-Hexadecyl-2-acetyl-glycero-3-phosphocholine	0.9	1.0
P_0012	1-Myristoyl-glycero-3-phosphocholine	1.1	0.9
P_0013	1-Palmitoyl-glycero-3-phosphocholine	1.0	0.9
P_0018	Sphingomyelin(d18:1/16:0)	0.8	0.8
N_0026	Cholesterol sulfate	0.9	0.9
P_0016	1-Stearoyl-glycero-3-phosphocholine	0.9	1.0
P_0019	Sphingomyelin(d18:1/18:0)	0.7	0.8
P_0008	Cholesterol	0.7	0.8
N_0007	Oleic acid	1.2	0.9
