# Supplementary material for: Transcriptome and Metabolome Analyses in Exogenous FABP4- and FABP5-Treated Adipose-Derived Stem Cells
Source: PLoS One. 2016 Dec 9;11(12):e0167825. doi: 10.1371/journal.pone.0167825 (PMC5148007; doi:10.1371/journal.pone.0167825)
Supplement: S9 Table — (PDF) [file pone.0167825.s018.pdf]

# S9 Table

Table S9. Primers for human genes in quantitative real-time PCR

| *Genes* | Accession # | Forward primer | Reverse primer |
|---|---|---|---|
| *18s* | M10098 | 5'- GTAACCCGTTGAACCCCATT -3' | 5'- CCATCCAATCGGTAGTAGCG -3' |
| *Myod1* | NM_000600 | 5'- CGGCATGATGGACTACAGCG -3' | 5'- CAGGCAGTCTAGGCTCGAC -3' |
| *Mef2a* | NM_002982 | 5'- GGTCTGCCACCTCAGAACTTT -3' | 5'- CCCTGGGTTAGTGTAGGACAA -3' |
| *Onecut1* | NM_005964.1 | 5'- GAACATGGGAAGGATAGAGGCA -3' | 5'- GTAGAGTTCGACGCTGGACAT -3' |
| *Jak3* | NM_006206.4 | 5'- TTCGGGCTACGCAAGGATTTG -3' | 5'- AGGCTGAGACACTCACCCT -3' |
